# ﻿Delineation of species of the *Tetramoriumcaespitum* complex (Hymenoptera, Formicidae) in Anatolia with a diagnosis of related species-complexes

**DOI:** 10.3897/zookeys.1234.142963

**Published:** 2025-04-22

**Authors:** Herbert C. Wagner, Marion Cordonnier, Bernard Kaufmann, Kadri Kiran, Celal Karaman, Roland Schultz, Bernhard Seifert, Sándor Csősz

**Affiliations:** 1 Centre for Ecological Research, Institute of Ecology and Botany, 2163 Vácrátót, Alkotmány u. 2-4, Hungary Institute of Ecology and Botany Vácrátót Hungary; 2 Institute of Biology, 8010 Graz, Universitätsplatz 2/I, Austria Institute of Biology Graz Austria; 3 Lehrstuhl für Zoologie/Evolutionsbiologie, Universität Regensburg, Universitätsstraße 31, D-93053 Regensburg, Germany Universität Regensburg Regensburg Germany; 4 Université Claude Bernard Lyon 1, LEHNA UMR 5023, CNRS, ENTPE, F-69622, Villeurbanne, France Université Claude Bernard Lyon 1 Villeurbanne France; 5 Department of Biology, Faculty of Sciences, Trakya University, 22030, Edirne, Turkiye Trakya University Edirne Turkiye; 6 Section Pterygota, Senckenberg Museum of Natural History, Am Museum 1, D-02826 Görlitz, Germany Senckenberg Museum of Natural History Görlitz Germany; 7 HUN-REN-ELTE-MTM Integrative Ecology Research Group, Pázmány Péter ave 1/C, Budapest 1117, Hungary HUN-REN-ELTE-MTM Integrative Ecology Research Group Budapest Hungary; 8 Department of Systematic Zoology and Ecology, Institute of Biology, ELTE-Eötvös Loránd University, Pázmány Péter ave 1/C, Budapest 1117, Hungary ELTE-Eötvös Loránd University Budapest Hungary

**Keywords:** Morphometrics, nest centroid clustering, microsatellites, pavement ants, *Tetramoriumflavidulum*, Türkiye

## Abstract

The high level of morphological crypsis of the hyper-diverse Palearctic *Tetramoriumcaespitum* group have challenged taxonomists for decades. Within this group, Wagner et al. (2017) offered a multidisciplinary solution for the delimitation of ten European species of the *Tetramoriumcaespitum* complex. Anatolia, harboring a high level of endemism in ants, has never been subject of focus research within this genus. In this study, the *Tetramoriumcaespitum* complex diversity in Anatolia and the Caucasus region was investigated by examining 191 nest-samples using an in-depth integrative-taxonomic approach. Quantitative morphometric and microsatellite data of 505 and 133 workers, respectively, and genital-morphology data of 33 nests were collected. Unsupervised analyses provided independent species-hypotheses based on the morphological and molecular disciplines. Based on the final species-hypotheses, we confirm *T.caespitum* (Linnaeus, 1758), *T.hungaricum* Röszler, 1935, *T.indocile* Santschi, 1927, *T.caucasicum*Wagner et al., 2017, *T.impurum* (Foerster, 1850), *T.immigrans* Santschi, 1927, and *T.flavidulum* Santschi, 1910 as valid species of the *T.caespitum* complex occurring in Anatolia. A lectotype of *T.flavidulum* was designated. The host of the temporary social-parasitic species *Tetramoriumaspina* Wagner et al., 2018 is *T.caucasicum* instead of *T.immigrans* – as it was suggested before. An identification key to species complexes of the *T.caespitum* group and to workers of the species of the *T.caespitum* complex in Anatolia is provided. Every cluster we identified could be linked to described species and the region’s species-composition is similar to those of the Balkans and Central Europe.

## ﻿Introduction

The genus *Tetramorium*, a diverse and ancient lineage with fossil records up to 37 million years old (Radchenko and Dlussky 2015), has diverged especially in the last 20 million years (Ward et al. 2015; Cicconardi et al. 2020). All native European and Anatolian *Tetramorium* species belong to the *Tetramoriumcaespitum* group (Bolton 1979; Kiran and Karaman 2012). However, within this group, the task of identifying not only species but even species complexes po­ses formidable challenges to taxonomists (Csősz and Schulz 2010; Wagner et al. 2017). Seven West-Palearctic species complexes have been outlined, each with its unique characteristics and intricacies: the *T.ferox* complex, the *T.chefketi* complex, the *T.caespitum* complex, the *T.semilaeve* complex (Csősz and Schulz 2010), the *T.striativentre* complex [as “*striativentre* species group”] (Radchenko and Scupola 2015), the *T.biskrense* complex [as “*biskrense* group”] (Lebas et al. 2016), and the *T.meridionale* complex [as “*Tetramorium meridionale* species-group”] (Salata et al. 2024). One further complex is briefly introduced in the frame of this study: the well-defined *T.inerme* complex with at least five species (*T.inerme* Mayr, 1877; *T.armatum* Santschi, 1927; *T.sulcinode* Santschi, 1927; *T.goniommoide* Poldi, 1979; *T.taueret* Bolton, 1995). In summary, we consider eight West Palearctic similar species-complexes within the *T.caespitum* group, each requiring a unique set of morphometric characters for delimitation.

The target complex of this study, the *Tetramoriumcaespitum* complex, has an age of approximately 6.78 million years (95% confidence interval: 8.66–2.23 million years) (Cicconardi et al. 2020). Of all complexes of the *T.caespitum* group, it goes farthest to the north, highest in altitude, and deepest into Siberia, so it includes the most oligothermic, frost resistant, and thus most widespread species in Central and North Europe (Steiner et al. 2010; Wagner et al. 2017; Seifert 2021). In Southern Europe and Anatolia, most species occur at high altitudes. The unexpected cryptic diversity in the *T.caespitum* complex was detected in the early 2000s (Steiner et al. 2002; Csősz and Markó 2004; Schlick-Steiner et al. 2006). The *T.caespitum* complex is monophyletic and crypsis is explained by morphological stasis (Wagner et al. 2018b). The in-depth taxonomic revision of Wagner et al. (2017) delimited ten European species, of which some hybridize (Cordonnier et al. 2019, 2020): *Tetramoriumalpestre*Steiner et al., 2010; *T.caespitum* (Linnaeus, 1758); *T.hungaricum* Röszler, 1935; *T.breviscapus*Wagner et al., 2017; *T.indocile* Santschi, 1927; *T.caucasicum*Wagner et al., 2017; *T.fusciclava* Consani & Zangheri, 1952; *T.staerckei* Kratochvíl, 1944; *T.impurum* (Foerster, 1850); and *T.immigrans* Santschi, 1927. In Siberia and East Asia, there are at least two further species: *Tetramoriumtsushimae* Emery, 1925 and *T.sibiricum* Seifert, 2021 (Steiner et al. 2006b; Seifert 2021). The taxonomic revision mentioned above (Wagner et al. 2017) mainly considered European but only very little Anatolian material because the predicted high number of cryptic species had discouraged the authors. Thus, a taxonomically unsatisfyingly solved situation in the diversity-hotspot Anatolia (cf. Kiran and Karaman 2012) remained.

The current study aims to delimit the Anatolian species of the *Tetramoriumcaespitum* complex based on an integrative-taxonomy approach. Morphological and molecular-genetic data are used as independent methods for establishing species hypotheses. Distribution and ecology data, and an identification key are provided.

## ﻿Materials and methods

### ﻿Integrative-taxonomy workflow

Species hypotheses given in Wagner et al. (2017), based on integrative taxonomy, were used as the starting hypotheses for the present study. With new specimens from Anatolia, we seek to untangle the intricate situation in the *T.caespitum* species complex. To this aim, our protocol for integrative taxonomy (Schlick-Steiner et al. 2010) is based on three methods, two of them quantitative and analyzed unsupervised (i.e., morphometrics and microsatellites) and one qualitative (i.e., male genital morphology). Mitochondrial DNA was not analyzed in this study, as it is of little value for species delimitation in the *Tetramoriumcaespitum* complex (Wagner et al. 2017) as well as in ants generally (Seifert 2018, 2024).

Artificial intelligence (AI) was not used in this study, but we will likely see large-scale deployment of this technology soon. The fact that morphometric data can separate the species of the *T.caespitum* complex makes them interesting candidates for testing some next-generation AI identification-techniques.

A workflow to assign new samples based on results of different disciplines was implemented as follows: A Nest-Centroid cluster, including all morphometric data of Anatolia and the Caucasus region, was established. Morphometric clusters were compared with male genital morphology and microsatellite data. Samples with discordant results between any disciplines were treated as wild cards in linear discriminant analyses (LDA) using morphometric data on the level of workers, performed with the software package SPSS Statistics v16 (IBM, USA) and the method “stepwise selection”, to fix species affiliation.

The Gene and Gene expression (GAGE) Species Concept (Seifert 2020, 1033) was employed in a conservative manner. It defines species as “… separable clusters that have passed a threshold of evolutionary divergence and are exclusively defined by nuclear DNA sequences and/or their expression products …”. This conservative use of the species concept was a deliberate choice, aimed at reducing the risk of over-splitting in this highly cryptic complex. Only species with at least two independent disciplines resulting in the same species-hypotheses were accepted (Schlick-Steiner et al. 2010), further ensuring the validity of our conclusions.

### ﻿Sampling

The study utilized material from 191 nest samples in Anatolia and the Caucasus region south of Russia. Among these, 168 samples were newly collected, while 23 were obtained from existing literature (Wagner et al. 2017; see Suppl. material 2). Since all available material was included, the distribution of investigated samples per species reflects their relative abundance in the field. The collected material was preserved in 96% ethanol. Additionally, material outside the *Tetramoriumcaespitum* complex was used to define the species complex within the *T.caespitum* group, following the taxonomic framework proposed by Bolton (1995). Distribution maps were created using QGIS Development Team (2019) based on our own data and relevant literature (Wagner et al. 2017).

### ﻿Morphometrics of workers

One worker per sample was used for DNA extraction. Three further individuals, if available, were mounted. If males were available, two workers and one male were prepared. In samples without males, three workers were prepared. If two workers were prepared, the largest and the smallest worker (evaluated by eye-estimation) of the sample were chosen. If three workers were prepared, the largest, the smallest, and one further worker of any size were prepared. This procedure aimed to cover extreme sizes to present a robust calibration set for discriminant analyses. Measurements were made using a Leica MZ16 A high-performance stereomicroscope with magnifications of ×80–296. Workers were positioned on a pin-holding stage permitting spatial adjustment in all directions. Measurements always referred to cuticula and not pubescence surface. An ocular micrometer with 120 graduation marks was used. Its measuring line was kept vertically to avoid the parallax error (Seifert 2002). A combination of a Fiberoptic L 150 light, equipped with two flexible light ducts, and a Leica KL 1500 LCD coaxial polarized light was used. All bilateral characters except PnHL (see definition) were measured from both sides and an arithmetic mean was calculated. Morphometric data of 505 workers from 191 nest samples were used (on average, 2.6 workers per sample). The used 31 characters were nearly identical as in Wagner et al. (2017); 26 of them originally go back to Csősz et al. (2014), Seifert (2007), or Steiner et al. (2006a). Ppss was modified to sqPpss, the square route of Ppss (used to transform data to normal distribution (as done for, e.g., PDCL in Seifert 2018)). Twenty-nine characters were collected morphometrically, MC1TG and POTCos meristically. The head index CS is a proxy measure for the size of individuals.

### ﻿Analyses of morphometric data

Nest-Centroid clustering (Seifert et al. 2014; Csősz and Fisher 2015) was used as unsupervised approach to establish morphological species-hypotheses independent from genetic data using R v3.0.1 and the packages MASS, ecodist, cluster, plyr, stringr, and scatterplot3d (Ligges and Maechler 2003; Goslee and Urban 2007; Wickham 2009, 2011; R Development core team 2012; Maechler et al. 2013; Ripley et al. 2013). Additionally, we employed a partitioning algorithm, Partitioning Based on Recursive Thresholding (Nilsen et al. 2013), using two distance metrics “part.kmeans” and “part.hclust” to estimate the ideal cluster-number and assign cases into partitions (clusters). The protocol was published by Csősz and Fisher (2016). NC clustering does often not allow for detecting species with only very few samples in the dataset but places them into the cluster of the next similar species. Thus, an alternative strategy was applied to detect rare species: In addition to the standard analyses, every sample was used as wild-card with available comparison data (Wagner et al. 2017) to detect putative samples of species known from the Balkans but not from Anatolia, for example, *T.staerckei*.

### ﻿Male genital morphology

Genital morphology of 33 males from 33 nests was qualitatively investigated. Mounted genitals of interest were used for z-stack imaging with a Keyence VHX-7000 digital microscope. All male genitals used for pictures are stored at the Senckenberg Museum of Natural History Görlitz. Representative images were used to draw anatomical figures. Interspecific differences of male genital morphology allowed a qualitative assessment in many cases.

**Figures 1–7.**
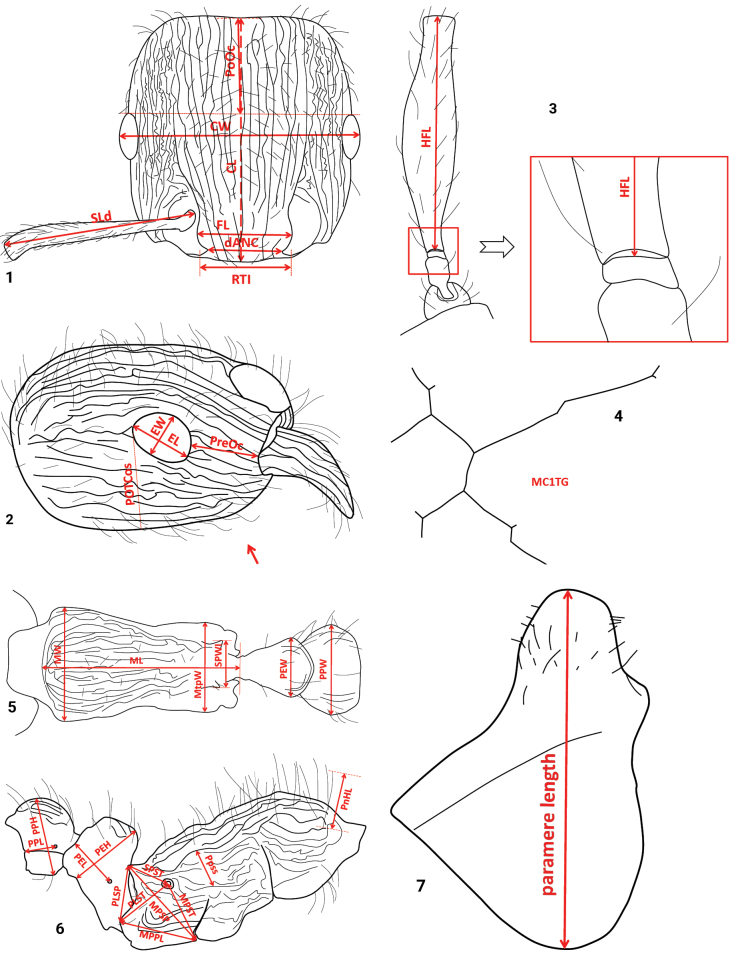
Measurement lines for the morphometric characters **1** CL, CW, dANC, FL, PoOc, RTI, and SLd **2** EL, EW, and PreOc; including an artificial line for the meristic character POTCos; in this example, POTCos = 7 **3** HFL **4** meristic character MC1TG. In this example, MC1TG = 18 **5** ML, MtpW, MW, PEW, PPW, and SPWI **6** MPPL, MPSP, MPST, PEH, PEL, PLSP, PLST, PnHL, PPH, PPL, Ppss, and SPST **7** paramere length (for abbreviations, see Table 1).

**Table 1.** Acronyms and definitions of the worker traditional morphometric characters and male paramere length slightly modified from Wagner et al. (2017). For illustrations, see Figs 1–6.

Acronym	Definition
CL	Maximum cephalic length in median line (Fig. 1); head is carefully tilted to position with true maximum; excavations of occiput and / or clypeus reduce CL. Peaks due to sculpture are ignored and only valleys are considered.
CS	Arithmetic mean of CL and CW.
CW	Maximum cephalic width across eyes (Fig. 1).
dANC	Minimum distance between antennal fossae (Fig. 1); measured in dorsofrontal view.
EL	Maximum diameter of one eye. All structurally defined ommatidia, pigmented or not, are included (Fig. 2).
EW	Minimum diameter of one eye. All structurally defined ommatidia, pigmented or not, are included (Fig. 2).
EYE	Arithmetic mean of EL and EW.
FL	Maximum distance between external margins of frontal lobes (Fig. 1). If this distance is not defined because frontal carinae constantly converge frontad, FL is measured at FRS level (definition of FRS see Seifert (2003)) as distance between the outer margins of frontal carinae.
HFL	Length of hind femur in dorsal view (Fig. 3). Second trochanter, which could appear to be portion of femur, must not be mistakenly included.
MC1TG	Quantification of stickman-like or reticulate microsculpture units on 1^st^ gastral tergite (use > 150× magnification; Fig. 4): Number of connected lines building units and being separated by line intersections and by flections angled > 10° is counted. Also very short lines are full counts. Straight lines twice as long as the typical length of one line, that is > 20 μm, are counted as 2. Arithmetic means of at least five units per worker are taken.
ML	Mesosoma length measured in dorsal view from caudalmost portion of propodeum to dorsofrontal corner of pronotal slope (i.e., where coarsely structured dorsum of pronotum meets finely structured pronotal neck; Fig. 5); equivalent measuring also possible in lateral view.
MPPL	Distance between most anterioventral point of metapleuron and most dorsocaudal point of propodeal lobe in lateral view (Fig. 6). If there are two points coming into question to be most dorsocaudal point on propodeal lobe, the one which is provided with a carina is taken.
MPSP	Distance between most anterioventral point of lateral metapleuron and distalmost point of propodeal spine (it does not need to be uppermost point of spine; Fig. 6).
MPST	Distance between most anterioventral point of metapleuron and center of propodeal stigma (Fig. 6).
MtpW	Maximum metapleuron width measured in dorsal view (Fig. 5). (In most cases, maximum is at caudal and in few cases at central or frontal region of metapleuron.)
MW	Maximum mesosoma width (Fig. 5).
paramere length	Maximum length of male genital paramere-structure in lateral view (Fig. 7).
PEH	Petiole height. Measured from uppermost point of concave ventral margin to node top (Fig. 6).
PEL	Petiole length. Measured in lateral view from center of petiolar stigma to caudal margin of petiole (both measuring points on same focal level; Fig. 6).
PEW	Maximum petiole width (Fig. 5).
PLSP	Distance between most dorsocaudal point of propodeal lobe (if there are two points coming into question to be most dorsocaudal point of propodeal lobe, the one which is provided with a carina is taken) and distalmost point of propodeal spine (it does not need to be uppermost point of spine; Fig. 6).
PLST	Distance between most dorsocaudal point of propodeal lobe and center of propodeal stigma (Fig. 6).
PnHL	Length of hair at frontolateral corner of pronotum (Fig. 6). Take longest hair of both sides.
PoOc	Postocular distance. Using cross-scaled ocular micrometer, head is adjusted to measuring position of CL; caudal measuring point: median posterior margin of head, microsculpture peaks are ignored and valleys are considered; frontal measuring point: median head crossing line between posterior eye margins (Fig. 1).
POTCos	Number of postoculo-temporal costae and costulae (Fig. 2). With head in lateral view and longitudinal axis of head adjusted horizontally, counted by focusing along perpendicular line from caudalmost point of eye down to underside of head. Costae / costulae just touching measuring line are counted as 0.5, those positioned just at ventral margin of head silhouette are not counted.
PPH	Maximum postpetiole height (Fig. 6).
PPL	Postpetiole length; distance from center of postpetiolar stigma to caudalmost intersection point of tergite and sternite (both measuring points at same focal level; Fig. 6).
Ppss	Maximum height of smooth and shiny area on lateral side of propodeum (Fig. 6). This area is brought into visual plane; a line is positioned perpendicular to main costae on propodeum and maximum height of smooth and shiny area without any costulae and costae is measured.
sqPpss	Square root of Ppss.
PPW	Maximum postpetiole width (Fig. 5).
PreOc	Preocular distance in lateral view. Measured as minimum distance between anterior eye margin and sharp frontal margin of gena (Fig. 2).
RTI	Distance between tops of ridges between antennal fossae and clypeus (Fig. 1). Tops are defined as most dorsofrontal points of ridges, provided with a costa on clypeus. Measured in dorso-anterior view.
SLd	Maximum scape length, including scape lobe, excluding articular condyle (Fig. 1).
SPST	Distance between distalmost point of propodeal spine (it does not need to be uppermost point of spine) and center of propodeal stigma (Fig. 6).
SPWI	Maximum distance between outer margins of propodeal spines (Fig. 5). Measured in dorso-anterior view.

### ﻿Microsatellite genotyping

DNA extraction from 170 whole individuals and the following microsatellite-genotyping protocols followed Cordonnier et al. (2018). For each microsatellite marker we computed the observed and expected heterozygosity, the number of alleles, and the effective alleles (GENALEX v. 6; Peakall and Smouse 2006) (Suppl. material 4). The identification of microsatellite clusters followed largely the procedure described in Cordonnier et al. (2018).

### ﻿Identification of microsatellite clusters

To determine the number of genetic clusters, we used the admixture model with correlated allele frequencies and with a number of a priori unknown clusters (*K*) varying from *K* = 1 to *K* = 20, running ten iterations for each *K*-value in the software STRUCTURE v. 2.3.1 (Pritchard et al. 2000). The dataset used included the 133 genotypes from Anatolia plus the genotypes of 12 individuals collected in France and Belgium (4 *Tetramoriumcaespitum*, 4 *T.immigrans*, and 4 *T.impurum*).

Following the procedure described in Cordonnier et al. (2018), each run of STRUCTURE consisted of 500,000 replicates of the MCMC after a burn-in of 500,000 replicates. The ten independent runs were analyzed with CLUMPAK (Kopelman et al. 2015) and the sets of similar runs grouped to generate a consensus solution for each distinct group. For each *K*, the different runs were either consensual, one single group of runs, or resulting in both a majority mode (larger part of the iterations) and minority mode(s) (remaining iterations). The software CLUMPAK allowed to identify the optimal *K*-value based on the median values of Ln(Pr Data) (Earl and von Holdt 2012). The membership coefficient of each individual at each of the *K* clusters corresponding to the consensus solution of the majority mode was selected as *Q*-value. Individuals were then assigned to a cluster based on their higher *Q*-value across the different clusters. Individuals having no *Q*-value higher than 0.6 were not assigned to any cluster.

### ﻿Reanalyzed linear discriminant-analysis (LDA) of morphometric data

After development of final species-hypotheses by the integrative-taxonomy approach, all nests were reanalyzed in a supervised approach using the same data as for Nest-Centroid clustering of morphometrics. SPSS Statistics v21 was used to perform the LDAs. To avoid overfitting, the number of individuals of each group had to be at least three times larger than the number of characters (Moder et al. 2007 and references therein).

### ﻿Thermal niches

Standard air-temperature (TAS) in °C, a rough approximation of the ecological niche (Seifert and Pannier 2007), was calculated as in Steiner et al. (2010). Only locality data from Wagner et al. (2017) and the current study were considered, all in all 165 species-locality combinations. TAS was used to explore ecological differences in re-analyses. TAS values were tested for species-specific differences using SPSS Statistics v. 16.0. Species-specific pairwise differences of TAS were calculated in SPSS 16.0 as 2-side independent-sample t-tests. Since in all cases Levene’s Test of Equality of Variances was > 0.05, *p* values for t-test type of line 1 (“variances are equal”) were accepted for Table 2. An α-level of 0.05 was used; in cases of multiple comparisons with single-character morphological distances, Bonferroni-Holm correction was applied (Holm 1979).

**Table 2.** Separation of species based on different methods: from left to right: NC clustering of morphometrics, male genital-morphology, and microsatellite analyses. Significant separations are signed with a checkmark, non-significant ones with a cross.

	*caespitum*			*hungaricum*			*indocile*			*caucasicum*			*impurum*			*immigrans*			*flavidulum*		
	NC cluster	male genital-morphology	microsatellite analysis	NC cluster	male genital-morphology	microsatellite analysis	NC cluster	male genital-morphology	microsatellite analysis	NC cluster	male genital-morphology	microsatellite analysis	NC cluster	male genital-morphology	microsatellite analysis	NC cluster	male genital-morphology	microsatellite analysis	NC cluster	male genital-morphology	microsatellite analysis
nests	17	2	11	11	3	7	19	4	9	47	14	23	25	3	17	48	1	32	24	5	17
*caespitum*																					
*hungaricum*	✓	✘	✘																		
*indocile*	✓	✓	✘	✓	✓	✓															
*caucasicum*	✓	✓	✘	✓	✓	✓	✓	✓	✘												
*impurum*	✓	✓	✓	✓	✓	✓	✓	✓	✓	✘	✓	✓									
*immigrans*	✓	✓	✓	✓	✓	✓	✓	✓	✓	✓	✓	✓	✓	✓	✓						
*flavidulum*	✓	✓	✓	✓	✓	✓	✓	✓	✓	✓	✓	✓	✓	✓	✓	✓	✓	✓			

### ﻿Type material

Type material of *Tetramoriumflavidulum* Santschi, 1910 belongs to the *Tetramoriumcaespitum* complex based on quantitative and qualitative evaluation of morphological data (details in Taxonomy).

## ﻿Results and discussion

### ﻿Morphometry

**Figure 8.**
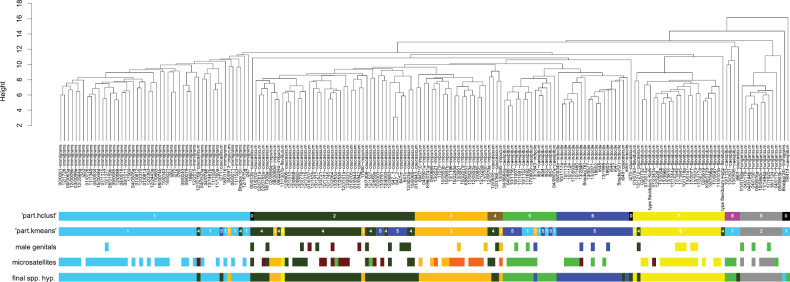
Nest-Centroid clustering of species occurring in Anatolia and Caucasus region. Results of both NC clustering algorithms “part.hclust” and “part.kmeans”, male genital morphology, microsatellites, and final species hypothesis are given in bars below the NC cluster.

The Nest-Centroid cluster showed seven separated large clusters (C1-7) including eleven to 50 nest samples each (Fig. 8; for morphometric data, see Suppl. material 2): C1: 46 samples of *T.immigrans*, 2 *T.caucasicum*, 1 *T.impurum*; C2: 37 *T.caucasicum*, 4 *T.impurum*, 1 *T.flavidulum*; C3: 18 *T.impurum*, 1 *T.caucasicum*; C4: 13 *T.caespitum*; 1 *T.indocile*; C5: 18 *T.indocile*, 1 *T.caucasicum*; C6: 23 *T.flavidulum*, 1 *T.caucasicum*; C7: 11 *T.hungaricum*. In C2, three samples of *T.impurum* build a subcluster within those of *T.caucasicum*. There are two smaller clusters: each with four samples, one with two of *T.caucasicum* and two of *T.impurum*, and one with three of *T.caespitum* and one of *T.caucasicum*. Moreover, there are four samples building clusters of their own, two of *T.caucasicum*, one of *T.immigrans*, and one of *T.caespitum*.

### ﻿Male genital morphology

We detected seven different male genital morphologies. Of them, six were already known (Wagner et al. 2017): Male genitals of *T.alpestre* sensu drawings in Wagner et al. (2017): detected in 3 samples of *T.caucasicum* (Suppl. material 1: fig. S1); *T.caespitum*/*hungaricum*: 4 *T.caespitum*, 3 *T.hungaricum*; *T.indocile*: 3 *T.indocile*; *T.caucasicum*: 11 *T.caucasicum*; *T.impurum*: 3 *T.impurum* (from east of 35° E); *T.immigrans*: 1 *T.immigrans*. Males of *T.indocile*, *T.impurum*, *T.immigrans*, and normal form of *T.caucasicum*, as already described in Wagner et al. (2017), can be delimitated at the species level with genital morphology. *Tetramoriumcaespitum* and *T.hungaricum* are the only species having identical male-genital-morphology. In addition to Wagner et al. (2017), we detected one new type of male genital morphology: The paramere structure of *T.flavidulum* (*n* = males of 5 nests) was very homogenous within the four samples of this species including males (Suppl. material 1: fig. S2). It belongs to the *impurum*-like form sensu Wagner et al. (2017) and is most similar with the normal form of *T.caucasicum* (Details under Treatment of species). The male genital morphology of western *T.caucasicum* samples (*n* = males of 3 nests from 3 sites), newly described here, is similar to those of *T.alpestre* but different from all Anatolian species as well as clearly different from eastern Anatolian and Caucasian *T.caucasicum*. The new *T.caucasicum* paramere structure belongs to the *T.impurum*-like form sensu Wagner et al. (2017): It has a rounded ventral paramere lobe without any corner in dorsal or ventral view but with clear division of ventral and dorsal paramere lobes, visible by deep emargination between lobes in posterior view. There is no sharp corner at the end of the ventral lobe visible in posterior view. The dorsal paramere lobe is relatively long and sharp-ended, visible in posterior and dorsal view. The ventral paramere lobe is slender than in *T.impurum*, visible in posterior view (Suppl. material 1: fig. S1). The paramere length of 3 males in lateral view was 956 ± 24 (928, 973) μm and thus below the range of *T.impurum*. Overall, there is no difference to *T.alpestre* but to all other species of the complex. Based on results of other disciplines, we consider western samples of *T.caucasicum* as conspecific but we cannot exclude that they might turn out to be a good species in future.

### ﻿Microsatellites

Bayesian clustering analysis based on microsatellite genetic data at 17 loci suggested either eight or ten distinct genetic clusters (*K* = 8: LnProb mean = -14410.237; *K* = 10: LnProb mean = -14462.500), but the mean similarity score between the runs of the major mode was higher for *K* = 8 (0.98 against 0.87 for *K* = 10). In view of parsimony, we therefore retained the simplest and more robust solution and considered 8 genetic clusters (Suppl. material 3).

Of 145 individuals, 116 Anatolian had *Q*-values > 0.6 and were considered for the analyses. The eight suggested clusters included samples of the following species (Fig. 8): Q1: 32 *T.immigrans*; Q2: 10 *T.caespitum*, 7 *T.indocile*, 2 *T.caucasicum*, and 1 *T.hungaricum*; Q3: 10 *T.caucasicum*, 1 *T.indocile*, and 1 *T.flavidulum*; Q4: 16 *T.flavidulum*; Q5: 12 *T.caucasicum* and 1 *T.impurum*; Q6: 7 *T.impurum* from west of 35° E; Q7: 10 *T.impurum* from east of 35° E; Q8: 6 *T.hungaricum*.

*Tetramoriumcaespitum* and *T.indocile* were not separated by this method while *T.caucasicum* and *T.impurum* were each split into two clusters. There were six further disagreements to morphological clustering.

### ﻿Integrative-taxonomy approach

Results of morphometrics, male genital morphology, and microsatellites largely concord (Fig. 8). Two samples of *T.caucasicum* are nested within the NC cluster of *T.immigrans*, which we explain by morphological crypsis. Several misclassifications occur in *T.caucasicum* and *T.impurum*. While these species are clearly separated from each other by male genital morphology and microsatellites, morphological similarity led to affiliation errors of four *T.impurum* samples nesting in the *T.caucasicum* cluster and one *T.caucasicum* nesting in the *T.impurum* cluster. Moreover, both species each include two microsatellite clusters, which we consider to represent intraspecific populations. In *T.caucasicum*, of seven samples with male genital plus microsatellite data available, the only one with genitals of the western form of *T.caucasicum* is also the only one of the microsatellite cluster Q3. The relation, however, is not significant (Fisher’s exact test, *p* = 0.1429). Hence, we consider the described differences as intraspecific variability. It has been already suggested that *Tetramorium* species of higher altitudes – due to the fragmented profile of mountains – are more difficult to identify because they might form local morphologi­cally and genetically distinct populations (Wagner et al. 2017). The new data of *T.caucasicum* are in line with this idea.

While eastern Anatolian males have an identical genital morphology as drawn in Wagner et al. (2017; *n* = 9 newly analyzed nests), western samples (*n* = 3) have male genitalia clearly different from the eastern but similar with the European species *T.alpestre* (Suppl. material 1: fig. S1). Conspecificity of western *T.caucasicum* samples with *T.alpestre*, however, can be rejected by worker morphometrics with the discriminant D_alp_ = 0.0269*SPWI+0.0447*MtpW+0.0727*dANC-0.0266*SLd-0.0170*PnHL+0.2220*sqPpss-0.0534*MPSP+0.0600*MPPL-0.1427*MC1TG-0.0996*EYE-1.9945. While western Anatolian *T.caucasicum* workers have values < 0 (error 2.9% in 35 workers and 0.0% in 12 nest means), *T.alpestre* workers have values > 0 (error = 0.0% in 142 workers). The geographic position of western Anatolian *T.caucasicum* between the main population of *T.caucasicum* in the Caucasus region and *T.alpestre* in Europe suggests that hybridization between these two alpine species could have occurred.

In *Tetramoriumimpurum*, we detected two separated lineages: Anatolian samples from west of 35° E and from Central Europe belong to the microsatellite cluster Q6 (Suppl. material 3); this line had been termed “*T.impurum* eastern clade” in the past (Wagner et al. 2017). Samples from east of 35° E belong to the microsatellite clade Q7, which was newly detected in the frame of this study. There is neither an NC-cluster difference (Fig. 8) nor a male-genital-morphology difference between these two lineages. However, the discriminant D_imp_ = -0.0385*HFL-0.0611*PPW+0.0601*PoOc+0.1046*FL-0.0300*RTI-0.1036*PreOc+0.1795*PPL+0.1142*MPPL-0.1127*PLST+0.1705*MC1TG-10.9419 separates 100% of Anatolian worker individuals (*n* = 36 workers from west of 35° E and 33 workers from east of 35° E). Workers from west of 35° E have values < 0, those from east of 35° E values > 0.

The species pair *T.caespitum* and *T.indocile* shows highest similarities in NC clusters with one *T.indocile* sample placed erroneously in the *T.caespitum* cluster. The Bayesian clustering approach we used did not allow separation of these two species based on microsatellites. A larger number of individuals or integration of a hierarchical approach (see, e.g., Balkenhol et al. 2014) could improve the delimitation of individuals from these genetically very similar species.

The NC cluster of *T.flavidulum* includes one sample of *T.caucasicum*, while, vice versa, the cluster of *T.caucasicum* also includes one sample of *T.flavidulum*.

To summarize, our integrative-taxonomy approach yielded evidence for seven clusters of nest samples for Anatolia: *Tetramoriumcaespitum* (17 samples), *T.hungaricum* (11), *T.indocile* (19), *T.caucasicum* (47), *T.impurum* (25), *T.immigrans* (48), and *T.flavidulum* (24).

### ﻿Reanalyzed linear discriminant-analysis (LDA) of morphometric data

For the reanalysis, 21 combinations for pairwise species comparisons were available. The mean error-rate of cross-validations of LDAs was 1.0%. Only one species pair had an error-rate higher than 5%: *T.caucasicum* and *T.indocile* with 5.7% (Table 3).

**Table 3.** Worker-individual error-rates of cross-validation LDA results for pairwise species or clade comparisons [%]. n = number of nests, i = number of individuals. Values < 5% in bold. (The number of individuals per group was at least 3× higher than the number of used characters.).

	*caespitum*	*hungaricum*	*indocile*	*caucasicum*	*impurum*	*immigrans*	*flavidulum*
n / i	17/42	11/30	19/43	47/132	25/69	48/113	24/76
*caespitum*							
*hungaricum*	**0.0**						
*indocile*	**2.4**	**0.0**					
*caucasicum*	**2.3**	**0.0**	5.7				
*impurum*	**0.0**	**0.0**	**0.9**	**5.0**			
*immigrans*	**0.0**	**0.0**	**0.0**	**2.0**	**0.5**		
*flavidulum*	**0.0**	**0.0**	**0.0**	**1.9**	**0.0**	**1.1**	

### ﻿Thermal niches

Species-specific ecological differences were significant in 14 of 21 pairwise species comparisons (67%) (Tables 4, 5). *Tetramoriumcaucasicum* had the lowest TAS values, followed by the three moderately thermophilous species *T.impurum*, *T.indocile*, and *T.caespitum*. Three species were distinctly thermophilous: *T.immigrans*, *T.hungaricum*, and *T.flavidulum* (Table 4).

**Table 4.** Standard air-temperature (TAS) comparison as an overview of ecological niches. Given are arithmetic means of localities ± standard deviation [lower extreme, upper extreme]; *n* = number of localities, TAS in °C. Only localities in Anatolia and the Caucasus region are considered.

species	*n*	TAS
*caespitum*	14	16.1 ± 2.8 [13.0, 23.2]
*hungaricum*	8	18.1 ± 1.3 [16.2, 20.4]
*indocile*	16	14.8 ± 1.8 [12.4, 19.6]
*caucasicum*	33	11.7 ± 2.3 [8.1, 17.5]
*impurum*	25	14.0 ± 2.2 [9.9, 18.9]
*immigrans*	46	19.2 ± 2.3 [15.1, 24.6]
*flavidulum*	23	17.1 ± 2.6 [12.1, 26.5]

**Table 5.** Species-specific standard air temperature. Significances at α = 0.05. Student’s t-test after Bonferroni-Holm correction are labeled with *.

Species	*caespitum*	*hungaricum*	*indocile*	*caucasicum*	*impurum*	*immigrans*	*flavidulum*
*caespitum*							
*hungaricum*	0.075						
*indocile*	0.127	< 0.001*					
*caucasicum*	< 0.001*	< 0.001*	< 0.001*				
*impurum*	0.010	< 0.001*	0.217	< 0.001*			
*immigrans*	< 0.001*	0.182	< 0.001*	< 0.001*	< 0.001*		
*flavidulum*	0.280	0.317	0.004*	< 0.001*	< 0.001*	0.001*	

### ﻿Type material assignment

**Figure 9.**
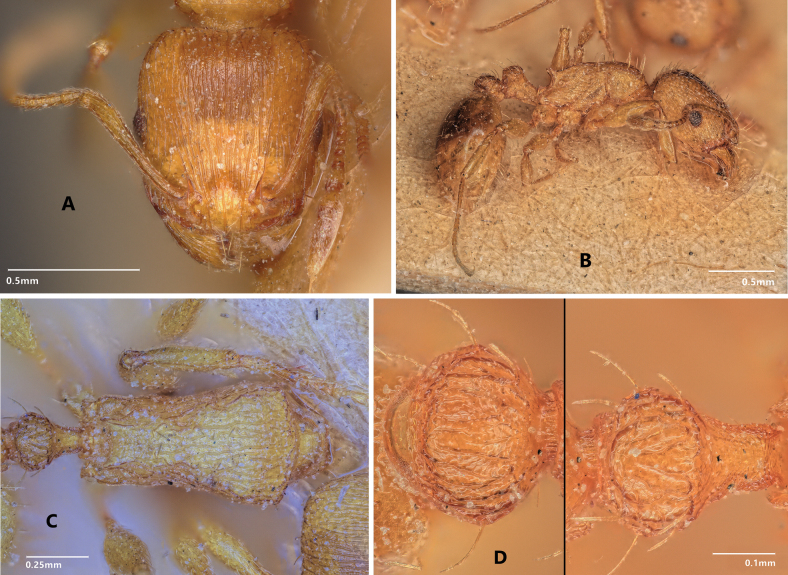
Lectotype of *Tetramoriumflavidulum* in (**A**) full face (**B**) lateral view. Paralectotype of *T.flavidulum* in dorsal view (**C** mesosoma, **D** petiole and postpetiole) (photographer RS).

Both type samples of *Tetramoriumflavidulum* fall into the NC cluster (Fig. 8) of the taxon which had been already considered to be *T.flavidulum* (Kiran and Karaman 2020). For the ten worker syntypes of “*Tetramoriumcaespitumflavidulum*“, collected by Max Korb between 1886 and 1900 (cf. Arnold 1921, D. 1933), using all morphometric variables the geometric mean was *p* = 1.00 in an 11-class LDA with wild-card run for the taxon (Fig. 9). Two syntype *T.flavidulum* workers, collected by Martin Holtz in 1897, using all morphometric variables, have a geometric mean of *p* = 0.96 for *T.caucasicum* and 0.03 for *T.caespitum*; including geographic coordinates *p* = 0.96 for *T.immigrans*, *p* = 0.02 for *T.flavidulum*, and 0.02 for *T.caespitum*. *Tetramoriumflavidulum* is the only species of the complex which can be identified by subjective characters quite well: Postpetiole with strong longitudinal costae, dorsum of petiole mostly strongly rugulose (Fig. 9D). Color often yellowish to light brown. MC1TG is high. Based on subjective investigation of morphology, types of both Korb and Holtz do not belong to any alternative species suggested by LDAs (*T.caespitum*, *T.caucasicum*, or *T.immigrans*). The ambiguous affiliation of the two type workers from Holtz, however, is unsatisfactory. We suggest that these types are untypical individuals of *T.flavidulum* but, since they are the only workers of the Anatolian south-coast used in this study, cannot fully exclude that they will turn out to belong to a cryptic species unknown to us and putatively with more southern distribution than the similar *T.flavidulum*. Thus, we have designated a worker of a card with 2 syntype workers, collected by Korb, as lectotype.

### ﻿The host of *Tetramoriumaspina* Wagner et al., 2018 is *T.caucasicum*

Three host workers of the type material of *T.aspina* (12/0859) in an 8-class LDA (including all Anatolian species and *T.staerckei*) using all morphometric variables belong to *T.caucasicum* with a geometric mean of *p* = 0.92, *p* = 0.04 to *T.immigrans*, and *p* = 0.04 for *T.flavidulum*. Including the three geographic variables they belong to *T.caucasicum* with a geometric mean of *p* = 0.99 and to *T.flavidulum* with *p* = 0.01. We found three further nests of *T.caucasicum* (including 2 nests with males showing the typical species-specific paramere-structure) and one of *T.impurum*, but none of *T.immigrans* syntopically. The TAS value of the site is 11.7, which is outside of the range of *T.immigrans* with 19.2 ± 2.3 [15.1, 24.6]. We conclude that this sample belongs to *T.caucasicum*. The misidentification of *T.caucasicum* as *T.immigrans* in Wagner et al. (2018a), detected in the frame of this study, resulted from an underestimation of the area of *T.caucasicum* into southwest and a lack of morphometric data from the southern part of its area (e.g., MC1TG was 21.4 and thus much higher than the species’ mean known at this time with 14.47 ± 1.81). In other words, it resulted from using an identification key for outside of the region for which it was designed. We can learn from this mistake that a large morphometric calibration background is needed before taxonomic conclusions can be drawn.

### ﻿Zoogeography

**Figure 10.**
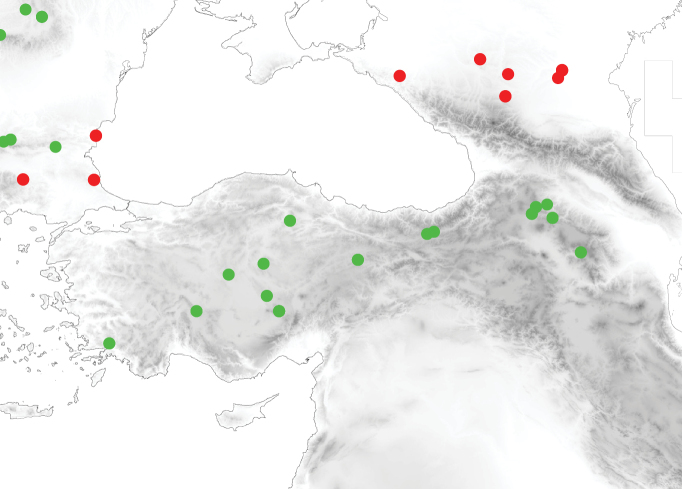
Distribution of *Tetramoriumcaespitum* (light green) and *T.staerckei* (red) in Anatolia and surrounding regions.

**Figure 11.**
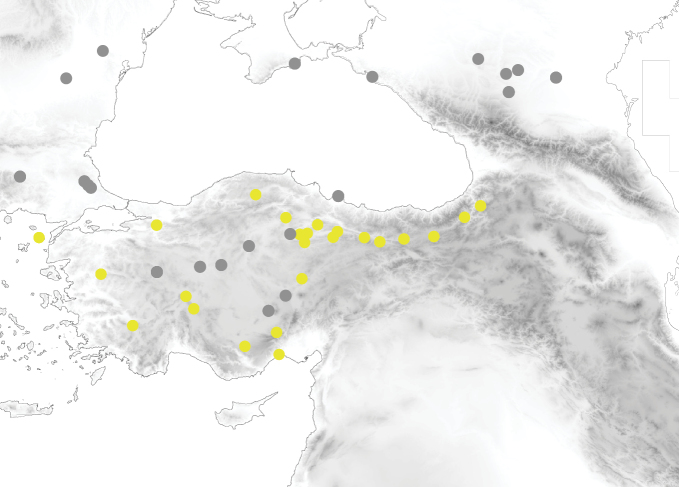
Distribution of *Tetramoriumhungaricum* (grey) and *T.flavidulum* (pale yellow) in Anatolia and surrounding regions.

**Figure 12.**
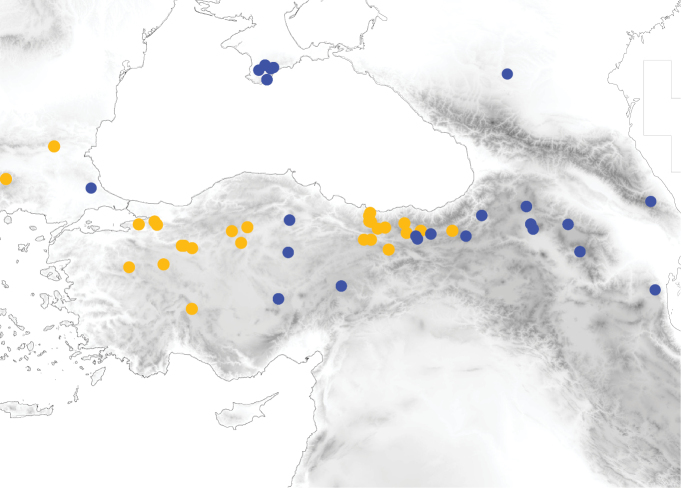
Distribution of *Tetramoriumindocile* (dark blue) and *T.impurum* (dark yellow) in Anatolia and surrounding regions.

**Figure 13.**
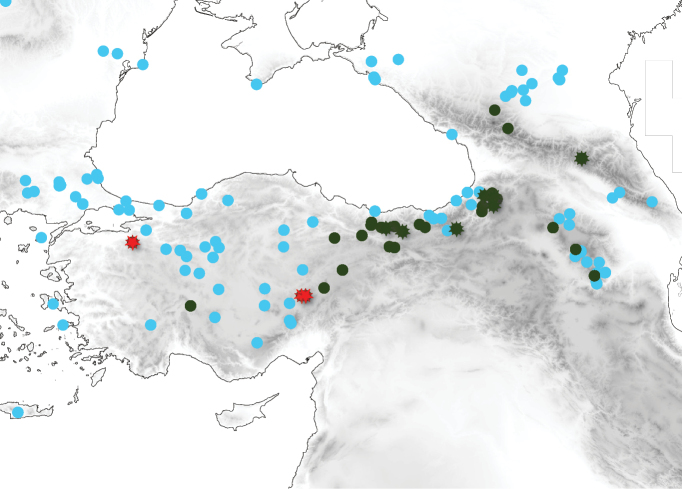
Distribution of *Tetramoriumcaucasicum* (dark green) and *T.immigrans* (cyan) in Anatolia and surrounding regions. Stars are records of *T.caucasicum* with males. Red stars show records with western (*alpestre*-like) and dark-green stars records with normal male genital morphology.

We demonstrated the occurrence of seven species of the *T.caespitum* complex in Anatolia (*T.caespitum*, *T.hungaricum*, *T.indocile*, *T.caucasicum*, *T.impurum*, *T.immigrans*, and *T.flavidulum*, Figs 10–13). Herewith, species diversity turned out to be lower than the authors had inferred before the results were available. Interestingly, the species composition in Anatolia is very similar as in Central Europe (with *T.alpestre*, *T.caespitum*, *T.hungaricum*, *T.indocile*, *T.staerckei*, *T.impurum*, and *T.immigrans*). Of Central European species, only *T.alpestre* and some COI clades of *T.caespitum* had (also) Western European or Apennine refugia; COI haplotypes of *T.caespitum* in eastern Central Europe, however, are more similar to those of the Caucasus than to those of the Apennine peninsula or western Europe (Wagner et al. 2017). *Tetramoriumhungaricum*, which is missing in Iberia and the Apennine peninsula, has a southeastern origin. *Tetramoriumindocile*, rare in Iberia and probably missing in the Apennine peninsula, might have originated in the Caucasus. *Tetramoriumimpurum* is absent from the Apennine peninsula and occurs in three genetically clearly different lineages (two of them described in Wagner et al. 2017). Its western clade occurs in Iberia and western Europe (Wagner et al. 2017; Attewell and Wagner 2019), the “eastern” clade in Central Europe, the Balkans, and western Anatolia; the latter has a southeastern origin and migrated from the Balkans or western Anatolia to Central Europe. A third clade, detected in the frame of this study, occurs in Anatolia east of 35° E. *Tetramoriumstaerckei*, a steppe species with an origin in southern Russia north of the Caucasus or Central Asia, migrated north of the Black Sea to the Balkans and Central Europe but not to Anatolia. In *Tetramoriumimmigrans*, a neozoon in Western and Central Europe (Gippet et al. 2017; Borowiec and Salata 2018; Seifert 2018; Castracani et al. 2020; Cordonnier et al. 2020; Sheard et al. 2020), high haplotype-diversity in mitochondrial DNA suggested Anatolia and the Caucasus region are the most likely geographic origin of *T.immigrans* (Wagner et al. 2017). *Tetramoriumflavidulum*, also of Anatolian or Caucasian origin, migrated northwest at least to Turkish Thrace (Bračko et al. 2016) and Greece (Finzi et al. 1928); Bulgarian records are doubtful (pers. comm. Albena Lapeva-Gjonova). We conclude that most Anatolian or Caucasian species migrated to Central Europe after the last ice age. Anatolia and the Caucasus region could also be the evolutionary origin of the species complex.

### ﻿Taxonomy

Diagnosis of the *Tetramoriumcaespitum* complex

1. Sexuals are larger than in most species outside the *Tetramoriumcaespitum* complex, MW of gynes > 1198 μm, CS > 1129 μm (but see, e.g., *T.moravicum*).
2. Gyne with high mesosoma in contrast to most species outside the *T.caespitum* complex.
3. Gyne with normal waist width and not with widened waist as species of the *T.ferox* complex or *T.meridionale* complex.
4. Male genital structure larger (paramere length > 843 μm) and with more species-specific features than in other complexes.
5. Males with ten antennal segments (not nine as in *T.biskrense* complex).
6. Some workers at underside of head with long c-shaped, crinkly, or sinuous hairs arising just behind buccal cavity (which are absent in most species outside the *T.caespitum* complex, but also present in *T.pelagium* Mei, 1995, *T.goniommoide* Poldi, 1979, and *T.feroxoides* Dlussky & Zabelin, 1985).
7. Workers without dense and distinct longitudinal striato-punctated sculpture on 1 ^st^ gastral tergite (as in the *T.striativentre* complex) but only stickman-like or reticulate microstructure with varying from few, scattered stickman-like to complex reticulate structures (Fig. 4), MC1TG < 33 (but not > 34 as in the *T.chefketi* complex).
8. Worker head, dorsum, and occiput with longitudinal costae and costulae, but occiput not with transversal or arching posterolaterally costae and costulae as in the *T.meridionale* complex.
9. Eye shorter than preocular distance, EL < PreOc (not as in most species of the *T.inerme* complex and partly in the *T.biskrense* complex where EL often > PreOc).
10. Metanotal groove shallow (not missing as in the *T.inerme* complex).
11. Propodeal spines short to medium but not reduced to small corners as in several species of the *T.ferox* (e.g., *T.aspina*) and the *T.inerme* complexes (e.g., *T.taueret*).
12. Worker head, mesosoma, petiole, and postpetiole surface partly smooth (as in *T.hungaricum* or *T.indocile*) to coarsely sculptured (as in *T.staerckei* or *T.flavidulum*) but not very coarsely sculptured as in the *T.chefketi* complex.
13. Color of most species brownish to blackish; in the Benelux, Central Europe, and Balkan mountain-areas sometimes light brown (*T.impurum*), in Anatolia even often yellowish (*T.flavidulum*).

The morphology of sexuals displays the most characteristic characters to define species complexes. Based on gyne morphology, we consider *Tetramoriumflavidulum* Santschi, 1910 as member of the *T.caespitum* complex. Gynes of the *T.ferox* complex differ from them by their wide waist (Csősz and Schulz 2010), those of the *T.chefketi* complex by the dense polygonal striation of the 1^st^ gaster tergite (Csősz et al. 2007), gynes of the *T.semilaeve* complex and the *T.inerme* complex are distinct smaller (Salata and Borowiec 2017).

### ﻿Taxonomic treatment by species

All Palearctic *Tetramoriumcaespitum* group names listed by Bolton (2014) have been evaluated recently concerning their possible affiliation to the *T.caespitum* complex (Wagner et al. 2017). Since then, no further West Palearctic species of the *T.caespitum* complex have been described (Bolton 2024). In addition to type material investigated by Wagner & al. (2017), it was necessary to investigate types of *T.flavidulum* Santschi, 1910:

*Tetramoriumflavidulum* Santschi, 1910 (12 workers of 2 samples) [Turkey]: 10 workers labeled as: “anatolia Korb” [—] MUSEO GENOVA coll. C. Emery (dono 1925) [—] SYNTYPUS „Tetramoriumcaespitumflavidulum“ [thereof we have chosen the lectotype worker]. 2 workers labeled as: “Tet. cespitum [sic!] v. flavidula [sic!] Em” [–] Asia minor Mersina 1897. Holtz [–] “Lectotype Tetramoriumflavidulum Emery, 1922” [–] “% designated by CSŐSZ, 2005” [–] MUSEO GENOVA coll. C. Emery (dono 1925) [–] ANTWEB CASENT0904803.

#### ﻿ Tetramorium flavidulum

Taxon classificationAnimalia

Santschi, 1910

D49CDCFA-BC4C-55F1-8A09-6063EBF11F96

##### Note.

Based on morphological criteria (Csősz et al. 2007; Csősz and Schulz 2010; Radchenko and Scupola 2015; Wagner et al. 2017; 2021) we place this species into the *Tetramoriumcaespitum* complex; however, we are unsure of its phylogenetic position. Future studies should investigate whether it is more closely related to species of the *T.caespitum* or the *T.chefketi* complex.

Tetramoriumcaespitumcaespitumvar.flavidula Emery, 1909: 702 (unavailable name); first available use: Tetramoriumcaespitumvar.flavidula Sant­schi, 1910; raised to species rank: Borowiec 2014: 198. Morphology of type material investigated.

##### Type locality.

Lectotype: Anatolia, leg. M. Korb, 1886–1900.

##### Lectotype designation.

Worker with non-decapitated body (of two syntype workers on one card; the other worker is decapitated with head fixed separately), labeled “anatolia Korb” [—] MUSEO GENOVA coll. C. Emery (dono 1925) [—] SYNTYPUS “Tetramoriumcaespitumflavidulum”. Lectotype worker and nine paralectotype workers in Museo Civico di Storia Naturale, Genova (Italy). Morphometric data of lectotype in μm: CL = 760, CW = 731, dANC = 199, EL = 152, EW = 110, FL = 285, HFL = 596, MC1TG = 29.6, ML = 845, MPPL = 250, MPSP = 331, MPST = 182, MtpW = 346, MW = 459, PEH = 256, PEL = 166, PEW = 240, PLSP = 160, PLST = 165, PnHL = 182, PoOc = 301, POTCos = 8.0, PPH = 253, PPL = 111, Ppss = 9, PPW = 283, PreOc = 197, RTI = 285, SLd = 595, SPST = 156, SPWI = 213.

##### Redescription of worker.

Medium size, CS = 734 ± 53 [614, 855]. 76% of workers with yellowish head and mesosoma and an often brownish gaster (which does usually not occur in other species of the complex), 21% of workers light to medium brownish, 3% dark brownish (*n* = 76 workers of 24 nests).

Head strongly elongate, CL / CW = 1.031 ± 0.018 [0.990, 1.088]. Eye medium-sized, EYE / CS = 0.176 ± 0.005 [0.167, 0.191]. Scape long, SLd / CS = 0.787 ± 0.015 [0.722, 0.815]. Mesosoma short and narrow, ML / CS = 1.110 ± 0.022 [1.057, 1.179], MW / CS = 0.619 ± 0.014 [0.580, 0.667].

Promesonotal dorsum convex, metanotal groove shallow. Head, dorsum, and occiput with longitudinal costae and costulae. Postoculo-temporal area of head with rather many costae and costulae, POTCos = 9.35 ± 2.47 [4.00, 15.50]. Mesosoma dorsum longitudinally rugulose, lateral side of propodeum with strongest sculpture of complex, Ppss = 15.7 ± 10.9 [6.0, 63.7]. Dorsum of petiolar node with strong reticulate costae, dorsum of postpetiole node with strong mostly longitudinal, sometimes reticulate costae. General surface appearance dull. Connected stickman-like or reticulate microsculpture: very large units scattered over 1^st^ gastral tergite, MC1TG = 25.93 ± 3.45 [16.30, 32.60]. Most workers with long c-shaped hairs on ventral head just posterior to buccal cavity, sinuous or crinkly hairs only in 13% of workers.

##### Description of male.

Yellowish. Ten antennal segments. Paramere structure belongs to the *impurum*-like form sensu Wagner et al. (2017). Ventral paramere lobe with one sharp corner visible in posterior view. Clear division of ventral and dorsal paramere lobes, visible by emargination between lobes in posterior view. Relatively short dorsal paramere lobe, visible in posterior and dorsal view. Maxi­mal paramere structure length in lateral view of four males 912 ± 27 (885, 949) μm. No corner on ventral paramere lobe between lobe top and emargination with dorsal lobe in dorsal and posterior view. Distinct different from all other species.

##### Distribution.

Known from 22 localities in Anatolia and Gökçeada Island (Fig. 11; more localities given in Kiran and Karaman 2020).

##### Ecology.

Rather thermophilic, TAS of 23 sites 17.1 ± 2.6 [12.1, 26.5]. More thermophilic than *T.indocile*, *T.caucasicum*, and *T.impurum*, less thermophilic than *T.immigrans*. 17 of 22 sites inhabiting woodland: *Quercus* forests (6), *Pinusnigra* forests (4), *Pinussylvestris* forest (1), *Pinussylvestris*-*Quercus* forests (2), other types of mixed forests (2), *Olea* stands (1), and scrublands (1). The rest in meadows (2), barren areas (1), river banks (1), and city centers (1).

##### Phenology.

Adult sexuals in nests on 2 July ± 12 [9 June, 13 July] (*n* = 7).

### ﻿Identification key to the complexes of the *Tetramoriumcaespitum* group

Data for this key have been taken from material investigated in the frame of this study and from the literature (Csősz and Schulz 2010; Borowiec et al. 2015; Radchenko and Scupola 2015; Lebas et al. 2016; Salata and Borowiec 2017; Wagner et al. 2017, 2018a, 2021).

**Table:** 

1	First gastral tergite, or at least its anterior half, with dense and distinct longitudinal striato-punctated sculpture. Asia only	***striativentre* complex** [see Dietrich 2004; Radchenko and Scupola 2015]
–	First gastral tergite without longitudinal striato-punctated sculpture but only stickman-like or reticulate microstructure	**2**
2	Occiput with transversal or arching posterolaterally costae and costulae. Mediterranean and Iran	***meridionale* complex** [Salata et al. 2024]
–	Occiput without transversal costae and costulae	**3**
3	Eye often longer than or with same length as preocular distance. No metanotal groove, propodeal spines short, and petiolar node dorsocaudally extended (Fig. 14). D_ine_: 0.0439*PPW+0.0105*FL+0.0409*SPST+0.0544*PreOc-0.0053*ML-0.0312*PEW-0.0221*MW-0.0663*EL+1.9998 < 0 (error 0.0% in 92 workers). Gynes smaller and with lower mesosoma than in *caespitum* complex. In Europe, only southern Russia and Caucasus; northern Africa and Asia	***inerme* complex**
–	Eye often shorter than preocular distance. D_ine_ > 0 (under exclusion of the three large-eyed western Mediterranean species *T.biskrense*, *T.pelagium*, and *T.fusciclava* error 2.3% in 1638 workers and 1.6% in 693 nest means; most errors in *T.semilaeve* complex and *T.hungaricum*)	**4**
4	Very complex stickman-like or reticulate microstructure on 1^st^ gastral ter­gite, MC1TG in 55 workers of species occurring in Europe > 34. If number of connected lines building units of stickman-like or reticulate microstructure smaller (*T.anatolicum* with MC1TG < 34), units are so dense that nearly connected with each other (but then not yellowish as *T.flavidulum*). All species except *T.anatolicum* very coarsely sculptured	***chefketi* complex** [see Csősz et al. 2007]
–	Microstructure on 1^st^ gastral tergite varying from few, scattered stickman-like to complex reticulate structures (Fig. 4), MC1TG < 33	**5**
5	Eye larger and/or distance between most anterioventral point of metapleuron and most dorsocaudal point of propodeal lobe larger, hind femur shorter and/or postpetiole lower. Discriminant D_bis_: 0.1210*EL+0.0726*MPPL-0.0357*HFL-0.0396*PPH-6.5356 > 0 (error 0.0% in 6 workers of *pelagium*, 5 of *brevicorne*, and 1 of *biskrense*). Males with only 9 antennal segments. Gynes smaller and with lower mesosoma than in *caespitum* complex. In Europe southern Spain, Corsica, Sardinia, Sicily, Lampedusa, and Linosa; common in North Africa	***biskrense* complex** [see Lebas et al. 2016]
–	Eye smaller and/or distance between most anterioventral point of metapleuron and most dorsocaudal point of propodeal lobe smaller, hind femur longer and/or postpetiole higher. D_bis_ < 0 (error 1.6% in 1648 workers and 0.5% in 733 nest means of *caespitum*, *ferox*, and *semilaeve* complex). Males with ten antennal segments	**6**
6	Discriminant D_fer_: 0.0157*CW-0.052*FL-0.069*PEW+0.074*PPH+1.4815 < 0 (error 2.6% in 461 workers and 1.1% in 93 nest means, Fig. 15). CS of gynes < 1113 µm (*n* = 78). Gynes with wide petiole and postpetiole, PEW / CS = 0.6115 ± 0.034, PPW / CS = 0.785 ± 0.040 (*n* = 22). Males small, paramere length < 843 μm. In Europe, Italy, Pannonia, and Balkans; Anatolia and Caucasus region	***ferox* complex** [see Csősz and Schulz 2010; Wagner et al. 2021]
–	D_fer_ > 0 (error 5.4% in 1990 workers and 2.3% in 794 nest means). Gynes with narrow or normal petiole and postpetiole (*semilaeve* complex: PEW / CS = 0.371 ± 0.014, PPW / CS = 0.492 ± 0.028, *n* = 26; *caespitum* complex: PEW / CS = 0.414 ± 0.032, PPW / CS = 0.607 ± 0.033, *n* = 54	**7**
7	Some workers at underside of head with long c-shaped, crinkly, or si­nuous hairs arising just behind buccal cavity. Discriminant D_sem_: 0.03096*CL-0.08355*FL+0.09060*PEW-0.07793*PPH-1.598 < 0 (error 6.4% in 1877 workers and 3.7% in 761 nest means, Fig. 16). Gynes large, CS > 1129 µm (*n* = 63). Gynes with high mesosoma. Males large, paramere length > 843 μm. Nearly whole Palearctic	***caespitum* complex** [see ‘Identification key to workers of the *Tetramoriumcaespitum* complex’ below]
–	C-shaped, crinkly, or sinuous hairs on underside of head absent. Discriminant D_sem_ > 0 (error 8.1% in 99 workers and 3.0% in 33 nest means). Gynes with low mesosoma. Males small, paramere length < 843 μm. Mediterranean	***semilaeve* complex** [see Csősz and Schulz 2010; Borowiec et al. 2015; Salata and Borowiec 2017]

**Figure 14.**
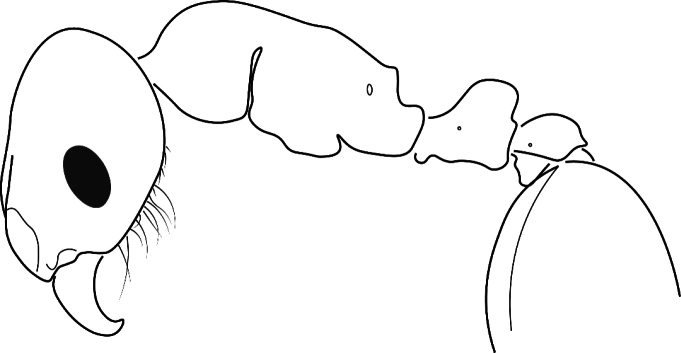
Schematic view of a *T.inerme* complex worker.

**Figure 15.**
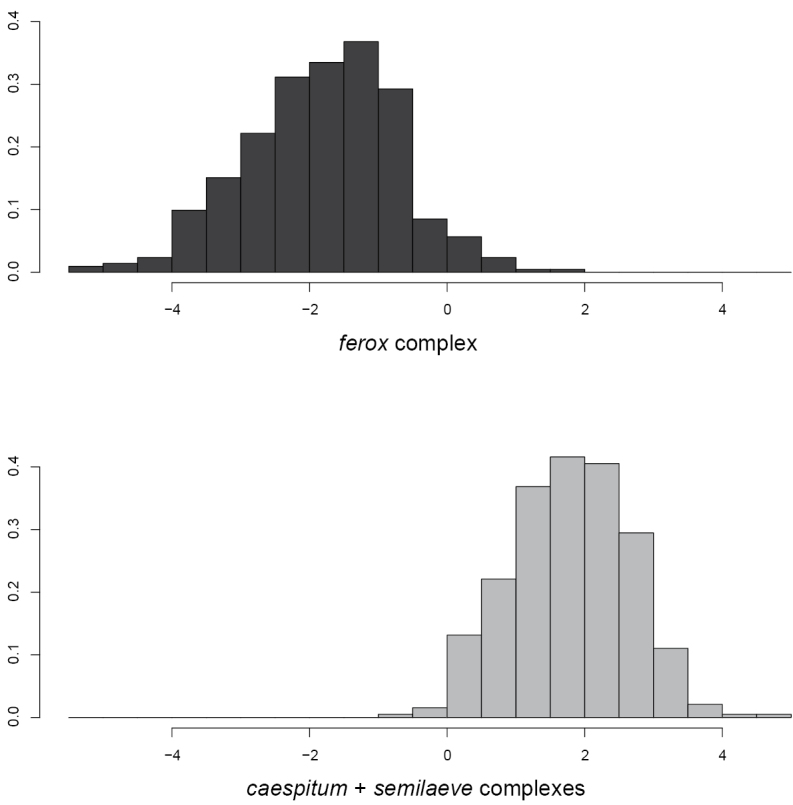
A linear discriminant-analysis separating workers of the *Tetramoriumferox* complex from those of the *T.caespitum* and *T.semilaeve* complexes.

**Figure 16.**
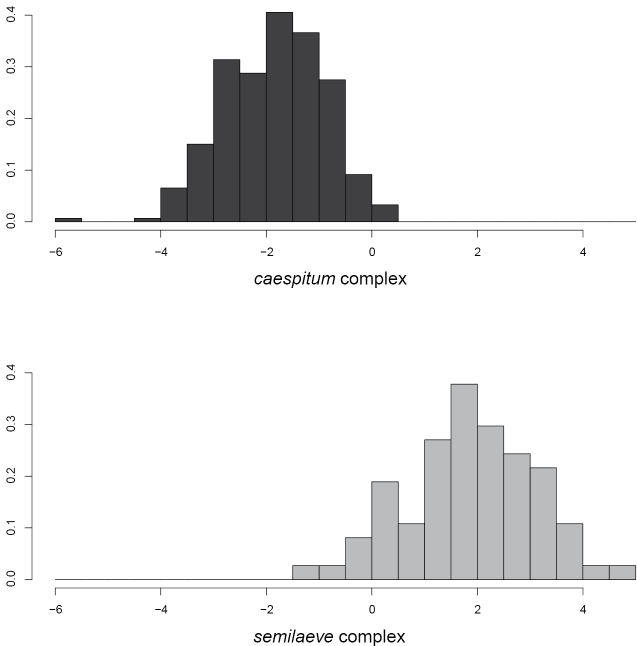
A linear discriminant-analysis separating workers of the *Tetramoriumcaespitum* complex from those of the *T.semilaeve* complex.

### ﻿Identification key to workers of the *Tetramoriumcaespitum* complex in Anatolia and the Caucasus

Data for this key have been taken from material investigated in the frame of this study and from the literature (Wagner et al. 2017); only data from Anatolia and the Caucasus region are included.

**Table:** 

1	Postpetiole with strong longitudinal costae (Fig. 9). Dorsum of petiole strongly rugulose. Stickman-like or reticulate microsculpture on first gastral strongly pronounced: MC1TG = 25.93 ± 3.45. Color often light: 76% of workers head and mesosoma yellowish and gaster yellowish to medium brownish, 21% of workers light to medium brownish, 3% dark brownish (*n* = 76 of 24 nests). Postpetiole narrow, short, and low: PPW / CS = 0.375 ± 0.014, PPL / CS = 0.145 ± 0.008, PPH / CS = 0.336 ± 0.013. Distances between center of propodeal stigma and most anterioventral point of metapleuron as well as most dorsocaudal point of propodeal lobe small: MPST / CS = 0.242 ± 0.007, PLST / CS = 0.239 ± 0.008. Discriminant D11: 0.0167*HFL+0.0118*ML+0.0149*MtpW-0.0356*PoOc-0.0436*FL+0.0301*dANC-0.0861*EL-0.0391*PreOc+0.0234*PEH+0.0289*PPH+0.0127*PnHL+0.1725*sqPpss-0.0521*MPSP+0.0922*MPST+0.0432*SPST-0.0503*MPPL-0.0241*PLSP-0.0897*MC1TG+6.8364 < 0 (error 3.9% of 76 workers and 4.2% of 24 nest means)	***flavidulum***
–	Postpetiole without strong longitudinal costae. Median dorsum of petiole fully smooth to strongly rugulose. Stickman-like or reticulate microsculpture on first gastral often less strongly pronounced. Usually, dark brown to blackish. Postpetiole often wider, longer, and higher: PPW / CS = 0.399 ± 0.018, PPL / CS = 0.155 ± 0.010, PPH / CS = 0.358 ± 0.014. Distances between center of propodeal stigma and most anterioventral point of metapleuron as well as most dorsocaudal point of propodeal lobe larger: MPST / CS = 0.259 ± 0.010, PLST / CS = 0.253 ± 0.011. D11 > 0 (error 1.9% in 429 workers and 0.0% of 166 nest means; most errors in *caucasicum*)	**2**
2	Sculpture on head and mesosoma reduced and large parts smooth and shiny. Very few postoculo-temporal costae and costulae: POTCos = 2.30 ± 1.77. Lateral face of propodeum anterior propodealstigma often smooth: Ppss = 88.01 ± 26.63. Petiole and postpetiole narrow, low, and short: PEW / CS = 0.296 ± 0.013, PEH / CS = 0.329 ± 0.008, PEL / CS = 0.220 ± 0.007, PPW / CS = 0.370 ± 0.013, PPH / CS = 0.337 ± 0.012, PPL / CS = 0.148 ± 0.011. Eye longer: EL / CS = 0.216 ± 0.008. Mesosoma short: ML / CS = 1.101 ± 0.022. Spines short: MPSP / CS = 0.401 ± 0.017, SPST / CS = 0.179 ± 0.011. Distance between most anterioventral point of lateral metapleuron and dorsocaudal point of propodeal lobe small: MPPL / CS = 0.327 ± 0.011. Small size: CS = 630 ± 51 µm. Discriminant D12: 0.0275*HFL+0.0291*SPWI-0.0307*SLd-0.119*POTCos+0.0818*EL-0.0356*PEL-0.0417*PPH+0.027*Ppss-0.0294*SPST+0.0524*MC1TG+1.7633 > 0 (error 0.0% of 30 workers)	***hungaricum***
–	Sculpture on head often more developed, extending over most parts of dorsal head surface. Number of postoculo-temporal costae and costulae higher: POTCos = 9.39 ± 3.08. Lateral face of propodeum anterior propodealstigma often not smooth: Ppss = 26.77 ± 18.82. Petiole and postpetiole wider, higher, and longer: PEW / CS = 0.321 ± 0.014, PEH / CS = 0.347 ± 0.011, PEL / CS = 0.231 ± 0.009, PPW / CS = 0.402 ± 0.016, PPH / CS = 0.359 ± 0.013, PPL / CS = 0.156 ± 0.010. Eye shorter: EL / CS = 0.196 ± 0.008. Mesosoma longer: ML / CS = 1.154 ± 0.031. Spines longer: MPSP / CS = 0.430 ± 0.020, SPST / CS = 0.197 ± 0.015. Distance between most anterioventral point of lateral metapleuron and dorsocaudal point of propodeal lobe larger: MPPL / CS = 0.342 ± 0.012. Often larger: CS = 748 ± 82 µm. D12 < 0 (error 0.3% of 399 workers and 0.0% of 155 nest means)	**3**
3	Sculpture well developed, number of postoculotemporal costae and costulae large, smooth area on lateral face of propodeum anterior propodeal stigma small: POTCos = 12.29 ± 2.16, Ppss = 21.3 ± 12.6. Eye wider: EW / CS = 0.153 ± 0.005. Distance between propodeal stigma and dorsocaudal end of propodeal lobe larger: PLST / CS = 0.262 ± 0.009. Hind femur longer: HFL / CS = 0.837 ± 0.023. D13: -0.0256*SPWI+0.0147*MtpW-0.0252*MW-0.0217*CL+0.0320*dANC+0.1281*POTCos+0.1427*EW-0.0428*EL+0.0492*PreOc+0.0202*PEH+0.0164*PPH+0.0133*PnHL-0.0954*MPSP+0.0216*PLST+0.0660*MPST+0.0725*SPST+0.1203*MC1TG-10.7343 > 0 (error 2.7% of 113 workers and 0.0% of 48 nest means)	***immigrans***
–	Sculpture strongly reduced to well developed. Eye narrower: EW / CS = 0.147 ± 0.006. Distance between propodeal stigma and dorsocaudal end of propodeal lobe smaller: PLST / CS = 0.251 ± 0.010. Hind femur shorter: HFL / CS = 0.795 ± 0.028. D13 < 0 (error 1.0% of 286 workers and 0.0% of 108 nest means)	**4**
4	Hind femur longer: HFL / CS = 0.829 ± 0.026. Mesosoma longer and wider: ML / CS = 1.189 ± 0.028, MtpW / CS = 0.502 ± 0.017, MW / CS = 0.647 ± 0.017. Postocular distance smaller: PoOc / CS = 0.389 ± 0.008. Often larger: CS = 782 ± 69. Stickman-like or reticulate microsculpture on first gastral tergite reduced: MC1TG = 10.88 ± 3.01. Distance between most anterioventral point of metapleuron and most dorsocaudal point of propodeal lobe larger: MPPL / CS = 0.353 ± 0.010. Distance between frontal carinae and ridges of frontal antennal fossae larger: FL / CS = 0.395 ± 0.008, RTI / CS = 0.407 ± 0.011. Postpetiole longer: PPL / CS = 0.165 ± 0.007. D14: -0.0256*HFL-0.0209*ML+0.0581*PEW-0.0482*MtpW+0.0685*PoOc+0.0869*EW-0.0374*PPL-0.0284*PPH-0.0111*PnHL+0.0563*MPST+0.1100*MC1TG+0.0228*MW-1.1852 < 0 (error 0.0% in 42 workers)	***caespitum***
–	Hind femur shorter: HFL / CS = 0.789 ± 0.024. Mesosoma shorter and narrower: ML / CS = 1.142 ± 0.027, MtpW / CS = 0.483 ± 0.014, MW / CS = 0.633 ± 0.014. Postocular distance larger: PoOc / CS = 0.408 ± 0.013. Often smaller: CS = 705 ± 55. Stickman-like or reticulate microsculpture on first gastral tergite moderate or pronounced: MC1TG = 16.61 ± 4.31. Distance between most anterioventral point of metapleuron and most dorsocaudal point of propodeal lobe smaller: MPPL / CS = 0.338 ± 0.012. Distance between frontal carinae and ridges of frontal antennal fossae smaller: FL / CS = 0.384 ± 0.011, RTI / CS = 0.391 ± 0.015. Postpetiole shorter: PPL / CS = 0.155 ± 0.010. D 14 > 0 (error 1.2% in 244 workers and 0.0% in 91 nest means)	**5**
5	Distance between antennae fossae larger: dANC / CS = 0.288 ± 0.008. Postocular distance smaller: PoOc / CS = 0.396 ± 0.010. Number of postoculo-temporal costae and costulae often smaller, POTCos = 5.82 ± 2.22. Stickman-like or reticulate microsculpture on first gastral tergite moderate: MC1TG = 13.11 ± 2.34. D15: 0.0182*SPWI+0.0429*MtpW+0.0511*PoOc-0.0819*dANC+0.0175*SLd+0.1641*POTCos+0.0407*PPL-0.0460*PPH-0.0610*SPST-0.0403*MPPL+0.0790*MC1TG+0.0335*PEH-0.0323*PEL-5.6482 < 0 (error 2.3% in 43 workers and 0.0% in 19 nest means)	***indocile***
–	Distance between antennae fossae smaller: dANC / CS = 0.277 ± 0.009. Postocular distance larger: PoOc / CS = 0.410 ± 0.012. Number of post­oculo-temporal costae and costulae often larger, POTCos = 8.30 ± 2.16. Stickman-like or reticulate microsculpture on first gastral tergite pronounced: MC1TG = 17.37 ± 4.27. D15 > 0 (error 3.0% in 201 workers and 1.1% in 91 nest means)	**6**
6	Distance between dorsocaudal end of propodeal lobe and propodeal spine as well as propodeal stigma larger: PLSP / CS = 0.233 ± 0.015, PLST / CS = 0.256 ± 0.009. Longest hair on frontolateral corner of pronotum longer: PnHL = 0.287 ± 0.028. D16: -0.0352*HFL+0.0257*ML-0.0554*MtpW+0.0327*MW+0.0296*SLd-0.1006*POTCos+0.0426*EL+0.0440*PreOc+0.0481*PPH-0.0210*PnHL-0.0389*SPST-0.0351*PLST-0.0296*PLSP+0.0739*MC1TG-0.0391*PEL < 0 (error 5.8% in 69 workers and 4.0% in 25 nest means)	***impurum***
–	Distance between dorsocaudal end of propodeal lobe and propodeal spine as well as propodeal stigma smaller: PLSP / CS = 0.215 ± 0.015, PLST / CS = 0.245 ± 0.010. Longest hair on frontolateral corner of pronotum shorter: PnHL = 0.260 ± 0.029. D16 > 0 (error 3.0% in 132 workers and 0.0% in 47 nest means)	***caucasicum***

## Supplementary Material

XML Treatment for Tetramorium flavidulum

## ﻿Additional information

### Author ORCIDs

Herbert C. Wagner https://orcid.org/0000-0002-5453-9357

Kadri Kiran https://orcid.org/0000-0001-7983-0194

Celal Karaman https://orcid.org/0000-0002-2158-5592

Bernhard Seifert https://orcid.org/0000-0003-3850-8048

Sándor Csősz https://orcid.org/0000-0002-5422-5120

### Data availability

All data generated or analyzed during this study are included in this published article and its supplementary information files.

## Supplementary materials

Supplementary material 1 Male genital morphology of species of the *Tetramoriumcaespitum* complex Data type docx Explanation note **fig. S1.** Male genital morphology of three males of *Tetramoriumcaucasicum* of western Anatolia in dorsal, ventral, lateral, and posterior view (photo­grapher RS, drawing by HCW). The genitals are very similar to those of *T.alpestre*. **fig. S2.** Male genital morphology of two males of *Tetramoriumflavidulum* of western Anatolia in dorsal, ventral, lateral, and posterior view (photographer RS, drawing by HCW). **fig. S3.** Male genital morphology of 3 males of *Tetramoriumalpestre* in dorsal, ventral, lateral, and posterior view (ex Wagner et al. 2017). **fig. S4.** Male genital morphology of 4 males of *Tetramoriumcaespitum* in dorsal, ventral, lateral, and posterior view (ex Wagner et al. 2017). **fig. S5.** Male genital morphology of 2 males of *Tetramoriumhungaricum* in dorsal, ventral, lateral, and posterior view (ex Wagner et al. 2017). **fig. S6.** Male genital morphology of 3 males of *Tetramoriumindocile* in dorsal, ventral, lateral, and posterior view (ex Wagner et al. 2017). **fig. S7.** Male genital morphology of 2 males of *Tetramoriumcaucasicum* in dorsal, ventral, lateral, and posterior view (ex Wagner et al. 2017). **fig. S8.** Male genital morphology of 2 males of *Tetramoriumstaerckei* in dorsal, ventral, lateral, and posterior view (ex Wagner et al. 2017). **fig. S9.** Male genital morphology of males of *Tetramoriumimpurum* in dorsal, ventral, lateral, and posterior view (ex Wagner et al. 2017). https://binary.pensoft.net/file/1313136This dataset is made available under the Open Database License (http://opendatacommons.org/licenses/odbl/1.0/). The Open Database License (ODbL) is a license agreement intended to allow users to freely share, modify, and use this Dataset while maintaining this same freedom for others, provided that the original source and author(s) are credited.Herbert C. Wagner, Marion Cordonnier, Bernard Kaufmann, Kadri Kiran, Celal Karaman, Roland Schultz, Bernhard Seifert, Sándor Csősz

Supplementary material 2 Morphometric data of workers of the *Tetramoriumcaespitum* complex in Anatolia and adjacent regions Data type xls https://binary.pensoft.net/file/1313137This dataset is made available under the Open Database License (http://opendatacommons.org/licenses/odbl/1.0/). The Open Database License (ODbL) is a license agreement intended to allow users to freely share, modify, and use this Dataset while maintaining this same freedom for others, provided that the original source and author(s) are credited.Herbert C. Wagner, Marion Cordonnier, Bernard Kaufmann, Kadri Kiran, Celal Karaman, Roland Schultz, Bernhard Seifert, Sándor Csősz

Supplementary material 3 Microsatellite *Q*-values of *Tetramoriumcaespitum* complex workers Data type xlsx https://binary.pensoft.net/file/1313138This dataset is made available under the Open Database License (http://opendatacommons.org/licenses/odbl/1.0/). The Open Database License (ODbL) is a license agreement intended to allow users to freely share, modify, and use this Dataset while maintaining this same freedom for others, provided that the original source and author(s) are credited.Herbert C. Wagner, Marion Cordonnier, Bernard Kaufmann, Kadri Kiran, Celal Karaman, Roland Schultz, Bernhard Seifert, Sándor Csősz

Supplementary material 4 Observed and expected heterozygosity, the number of alleles, and the effective alleles of microsatellite data Data type docx https://binary.pensoft.net/file/1313139This dataset is made available under the Open Database License (http://opendatacommons.org/licenses/odbl/1.0/). The Open Database License (ODbL) is a license agreement intended to allow users to freely share, modify, and use this Dataset while maintaining this same freedom for others, provided that the original source and author(s) are credited.Herbert C. Wagner, Marion Cordonnier, Bernard Kaufmann, Kadri Kiran, Celal Karaman, Roland Schultz, Bernhard Seifert, Sándor Csősz
