# Supplementary material for: ﻿Delineation of species of the Tetramoriumcaespitum complex (Hymenoptera, Formicidae) in Anatolia with a diagnosis of related species-complexes
Source: Zookeys. 2025 Apr 22;1234:309–39. doi: 10.3897/zookeys.1234.142963 (PMC12041867; doi:10.3897/zookeys.1234.142963)
Supplement: Supplementary material 1 — Male genital morphology of species of the Tetramoriumcaespitum complex [file zookeys-1234-309_article-142963__-s001.docx]

Supplement Figs S1-S9 of

**Delineation of species of the *Tetramorium caespitum* complex (Hymenoptera, Formicidae) in Anatolia with a diagnosis of related species-complexes**

Herbert C. Wagner, Marion Cordonnier, Bernard Kaufmann, Kadri Kiran, Celal Karaman, Roland Schultz, Bernhard Seifert, Sándor Csősz


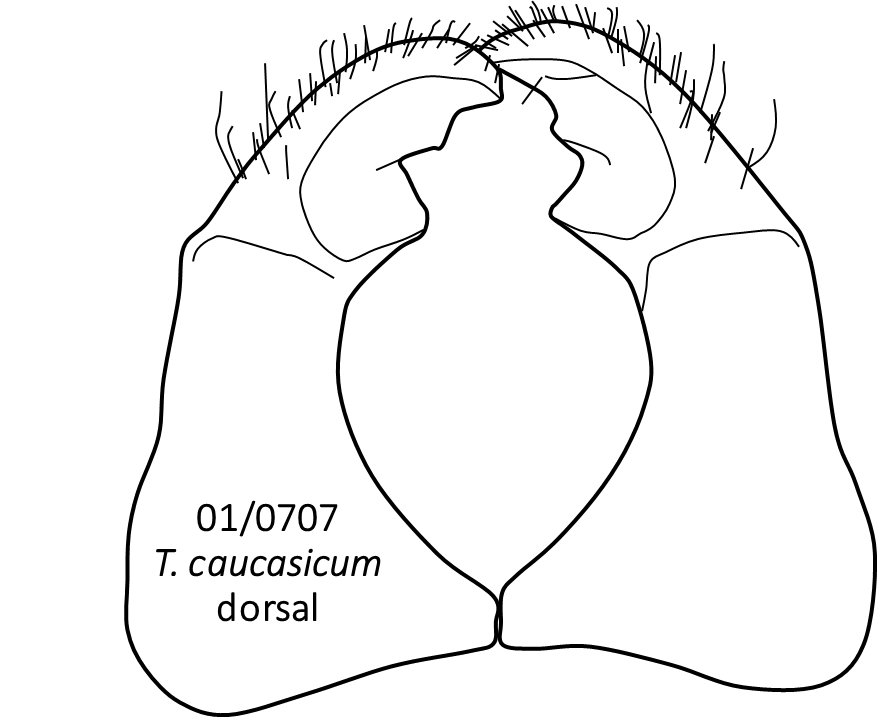

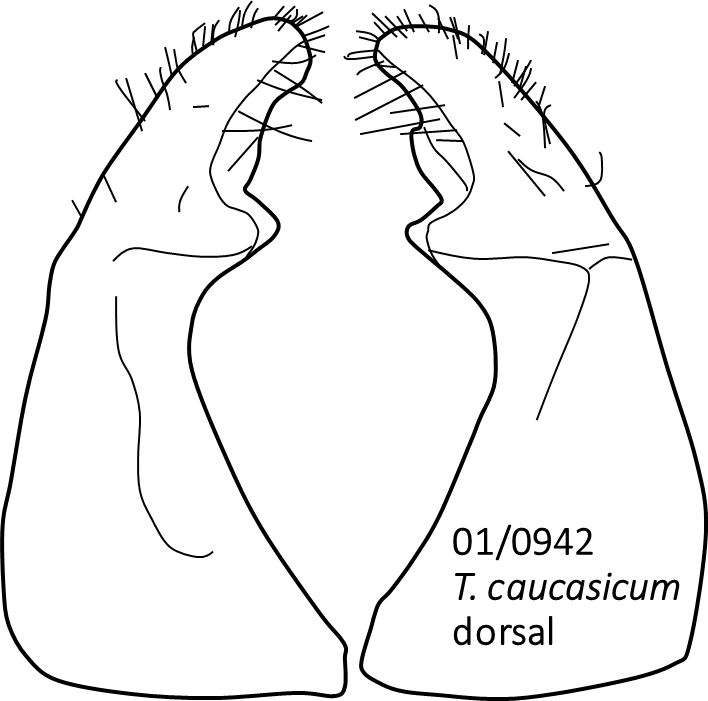

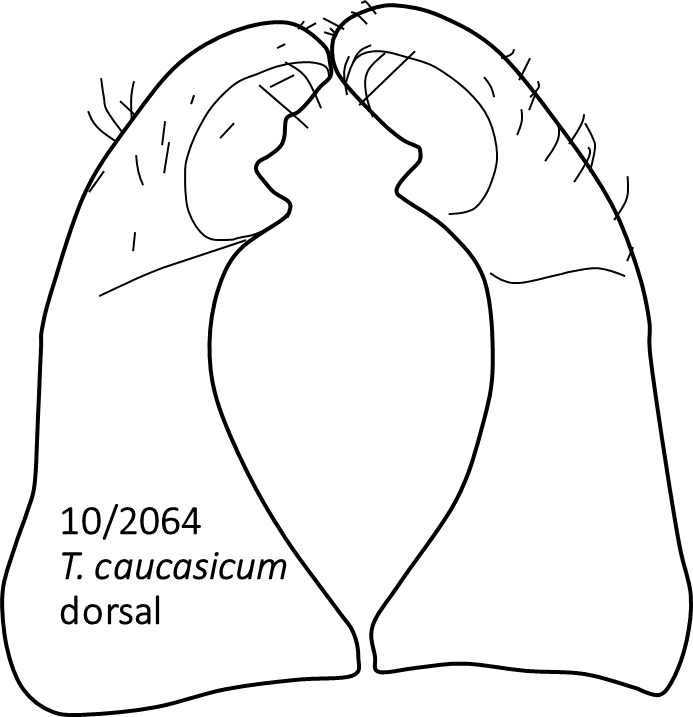


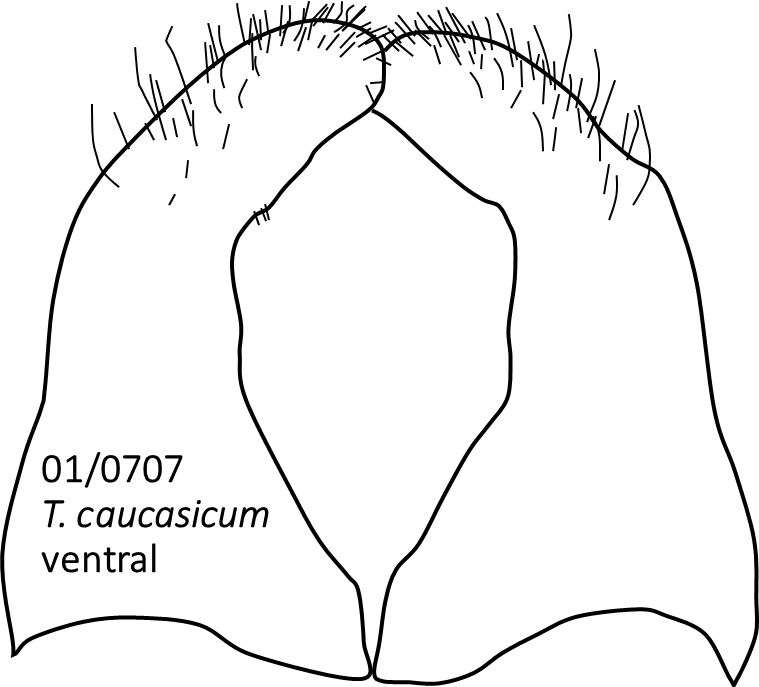

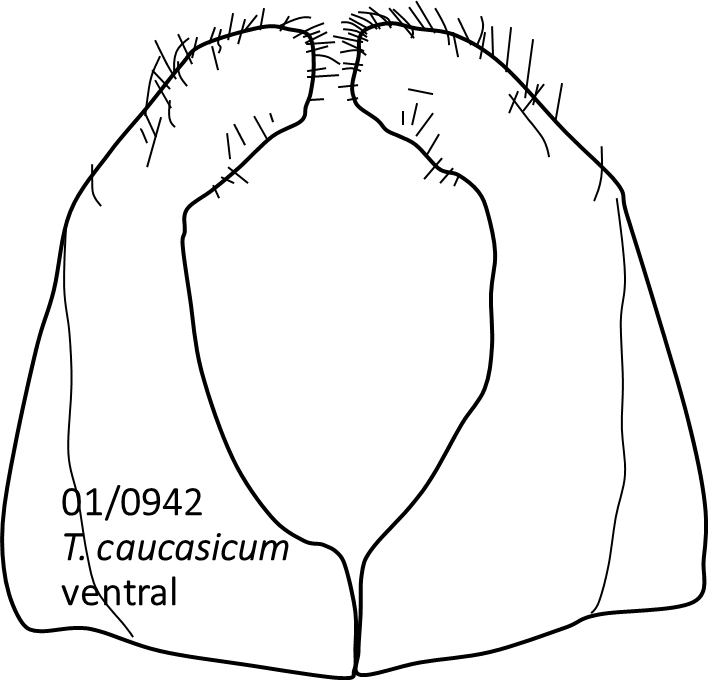

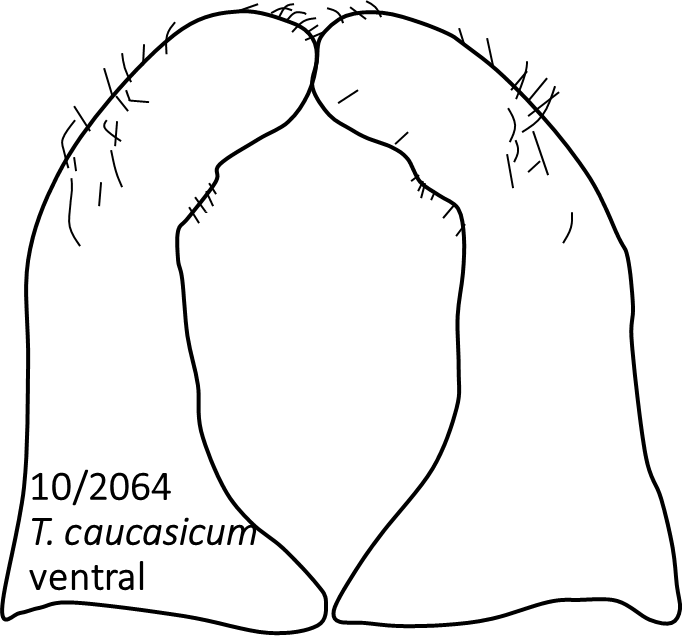


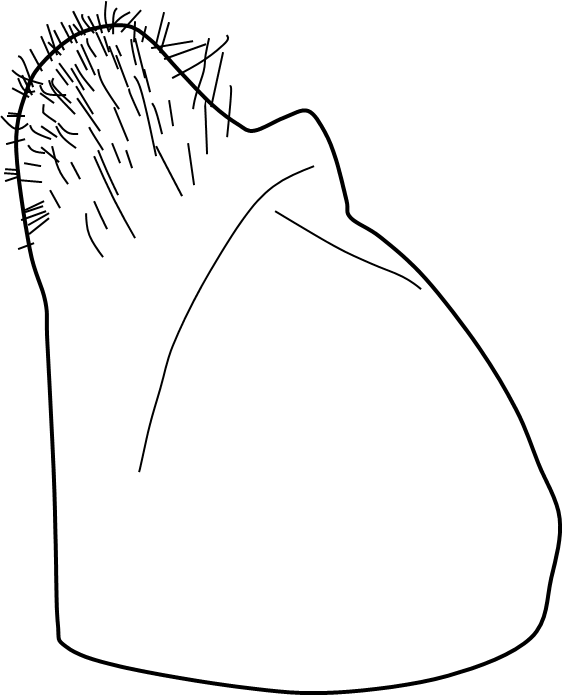

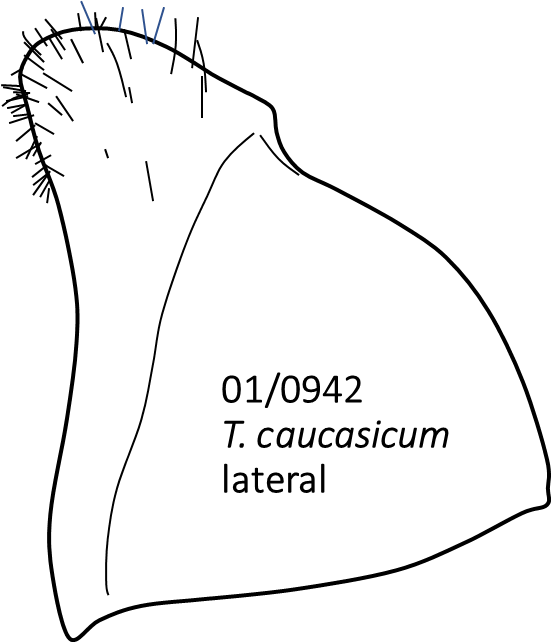

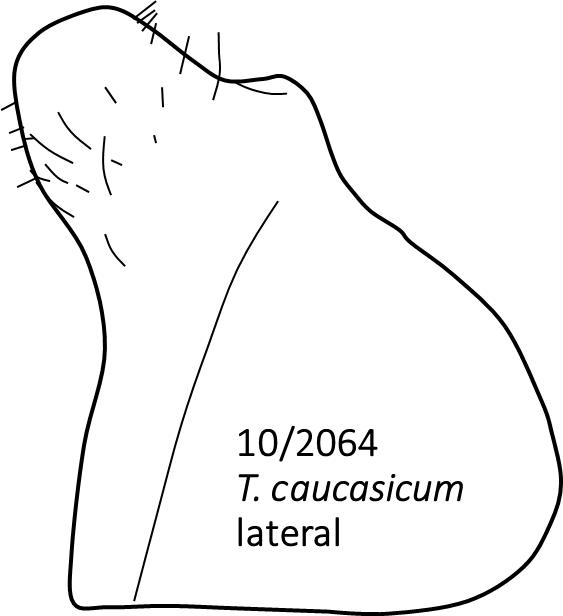


**01/0707
*T. caucasicum*lateral**


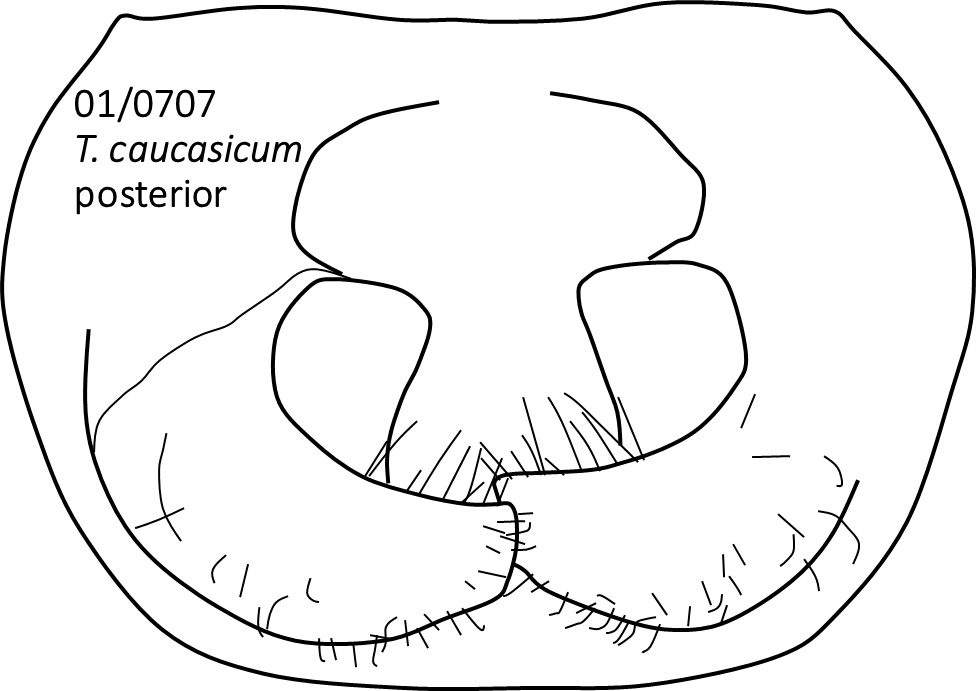

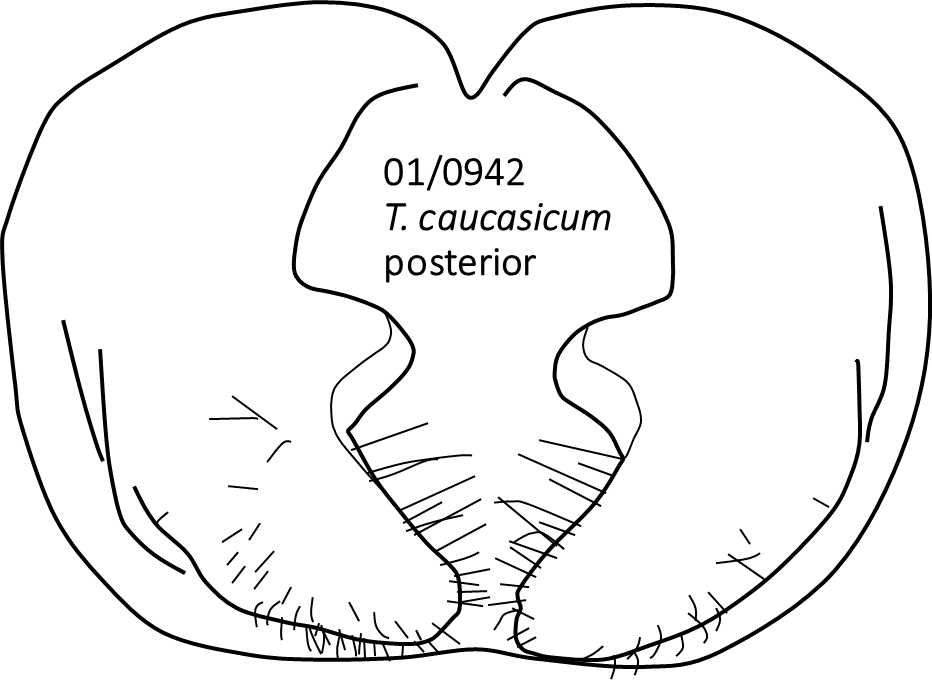

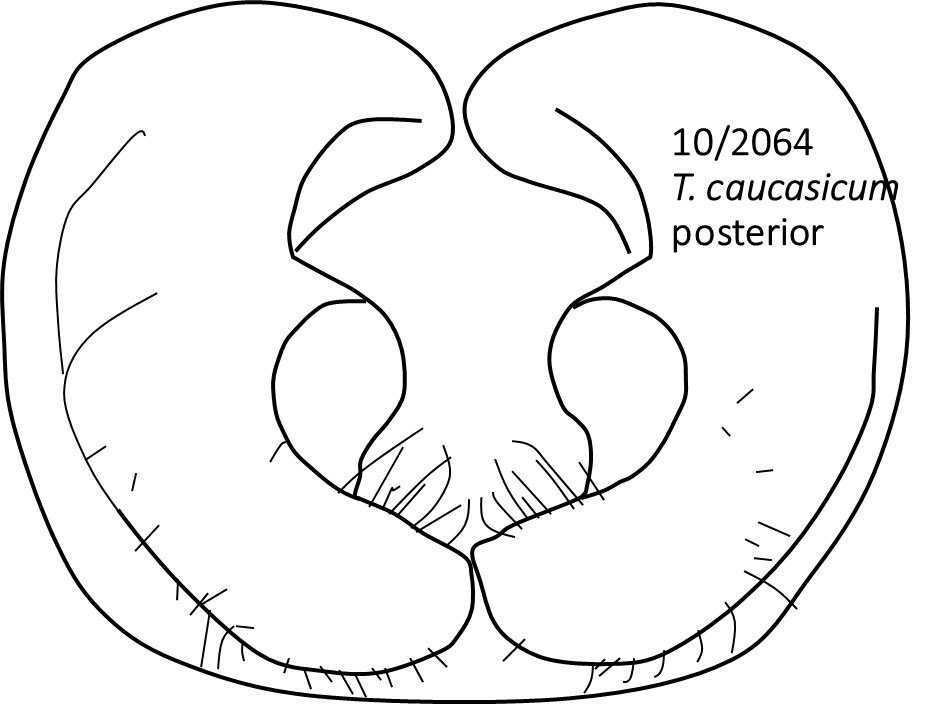


Fig. S1: Male genital morphology of 3 males of *Tetramorium caucasicum* of western Anatolia in dorsal, ventral, lateral, and posterior view (photographer Roland Schultz, drawing by Herbert C. Wagner). The genitals are very similar to those of *T. alpestre*.


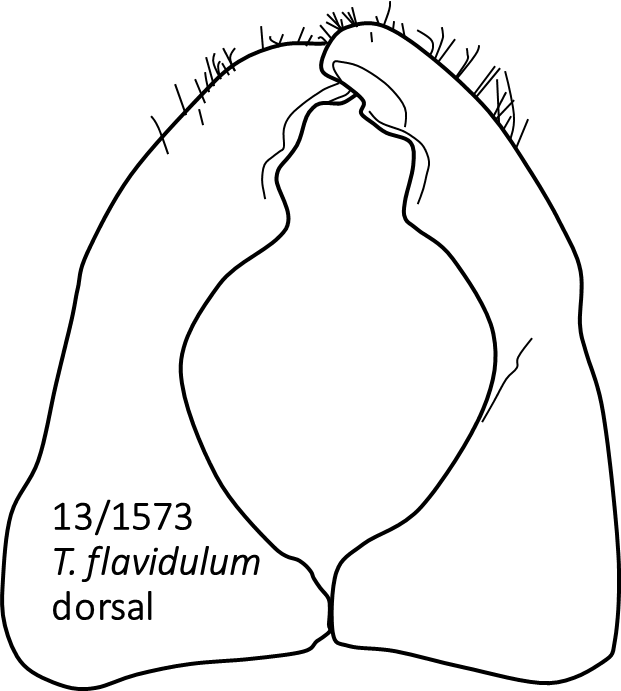

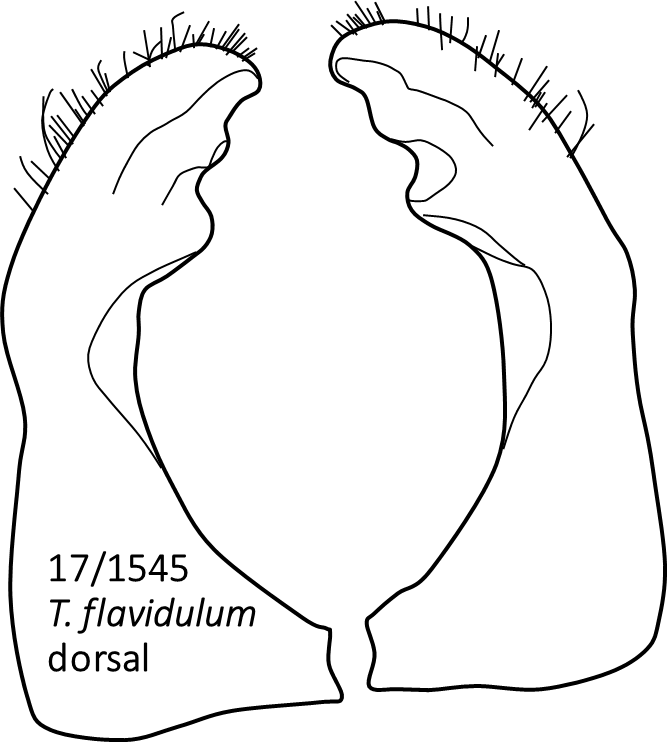


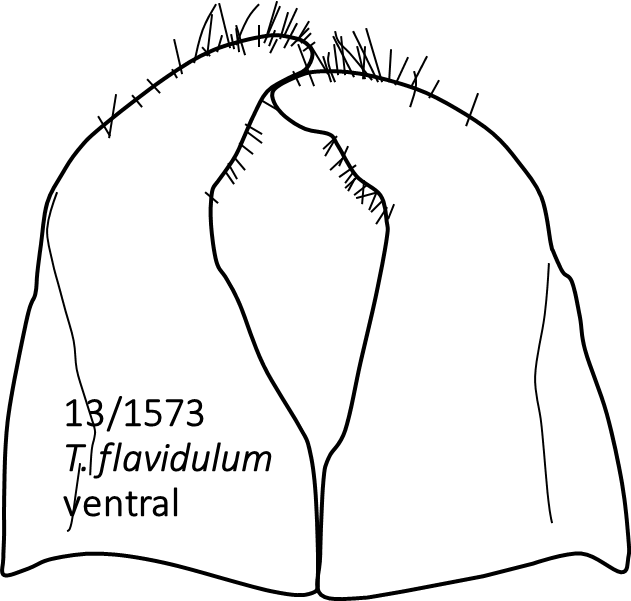

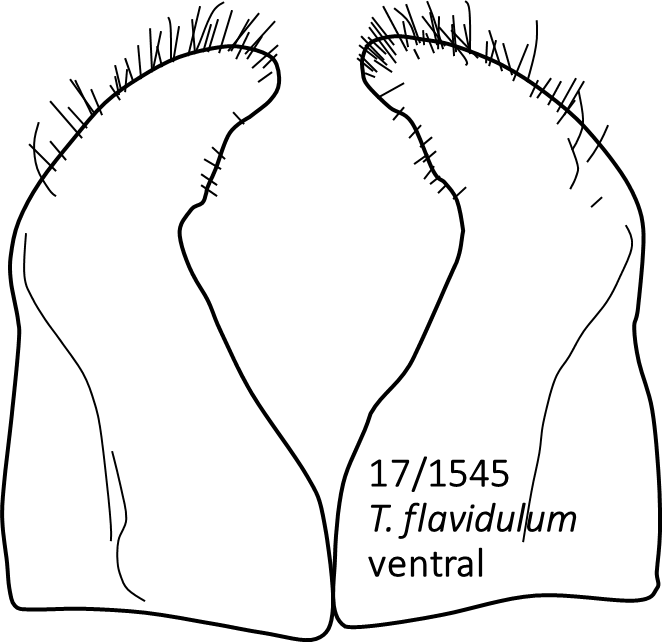


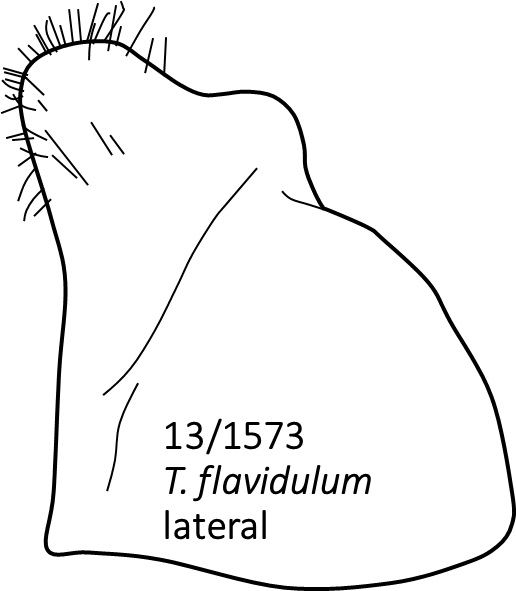

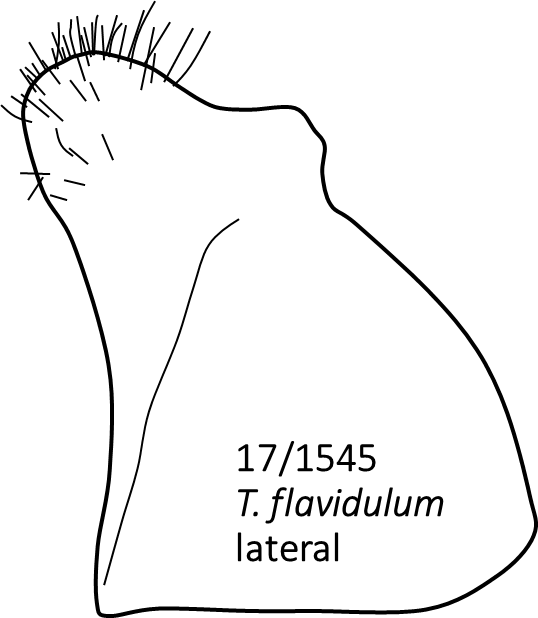


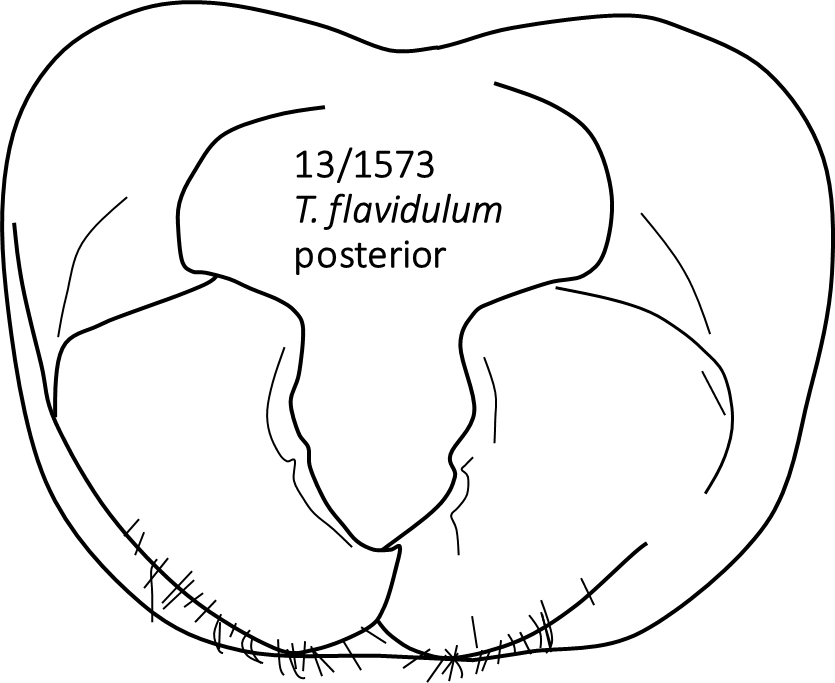

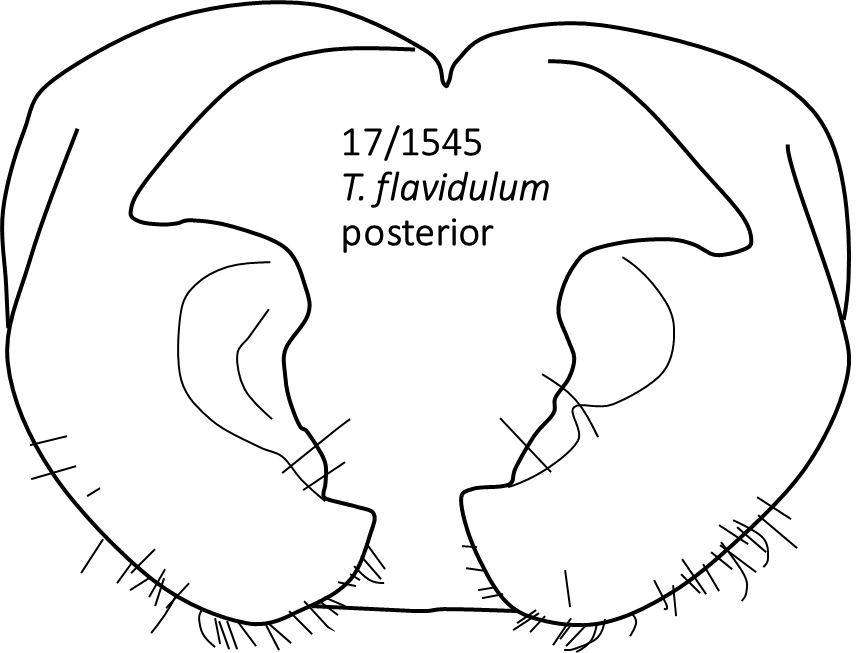


Fig. S2: Male genital morphology of 2 males of *Tetramorium flavidulum* of western Anatolia in dorsal, ventral, lateral, and posterior view (photographer Roland Schultz, drawing by Herbert C. Wagner).


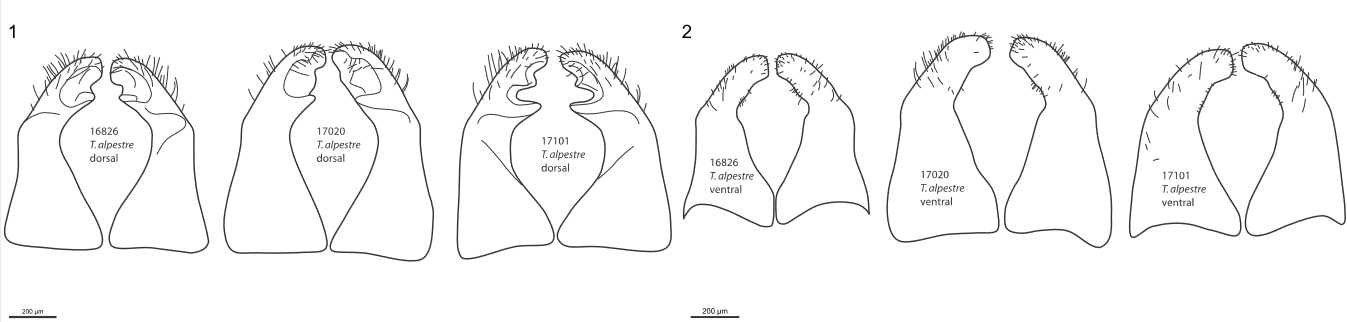

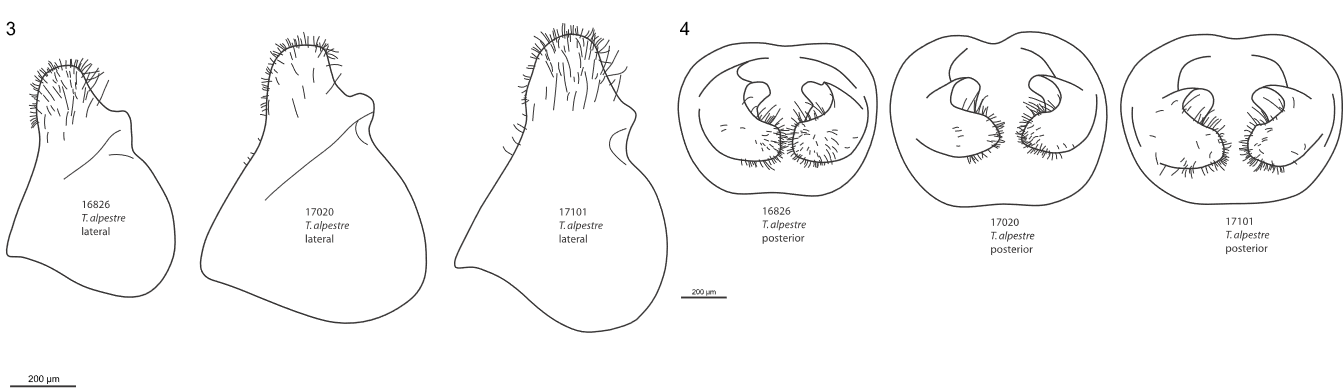


Fig. S3: Male genital morphology of 3 males of *Tetramorium alpestre* in dorsal, ventral, lateral, and posterior view (ex Wagner et al. 2017).


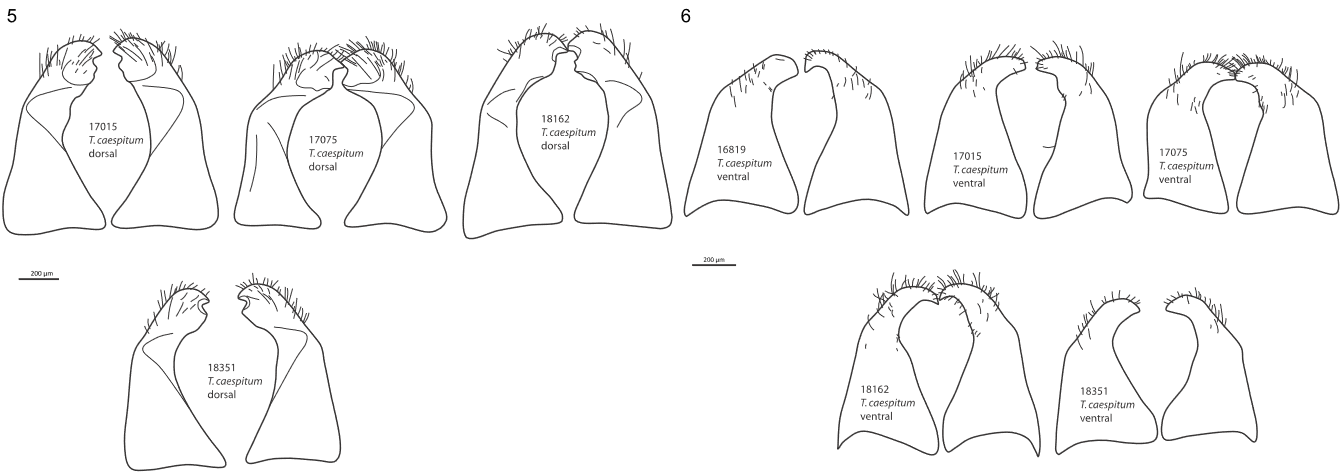

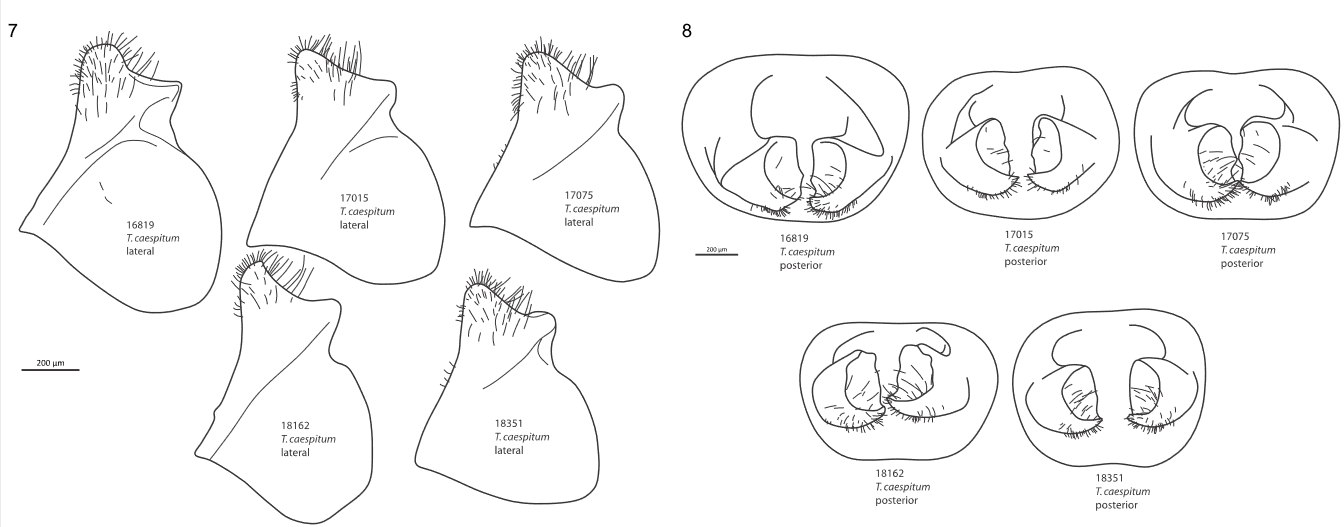
Fig. S4: Male genital morphology of 4 males of *Tetramorium caespitum* in dorsal, ventral, lateral, and posterior view (ex Wagner et al. 2017).


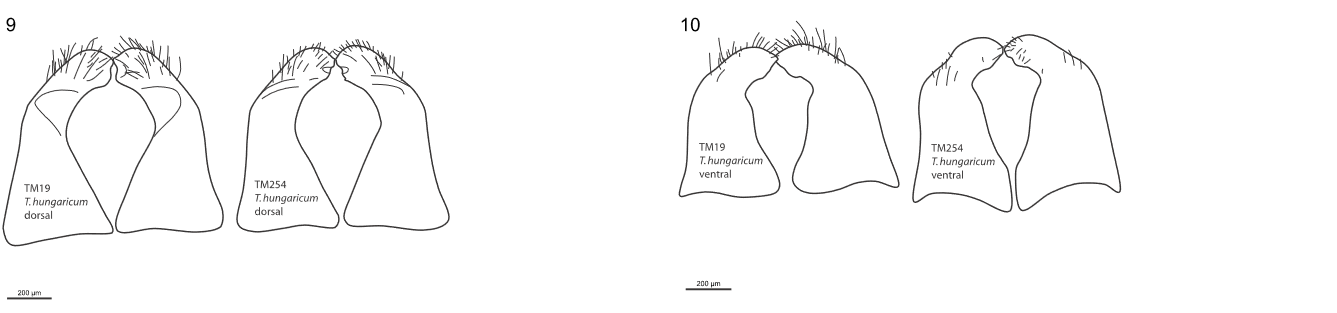

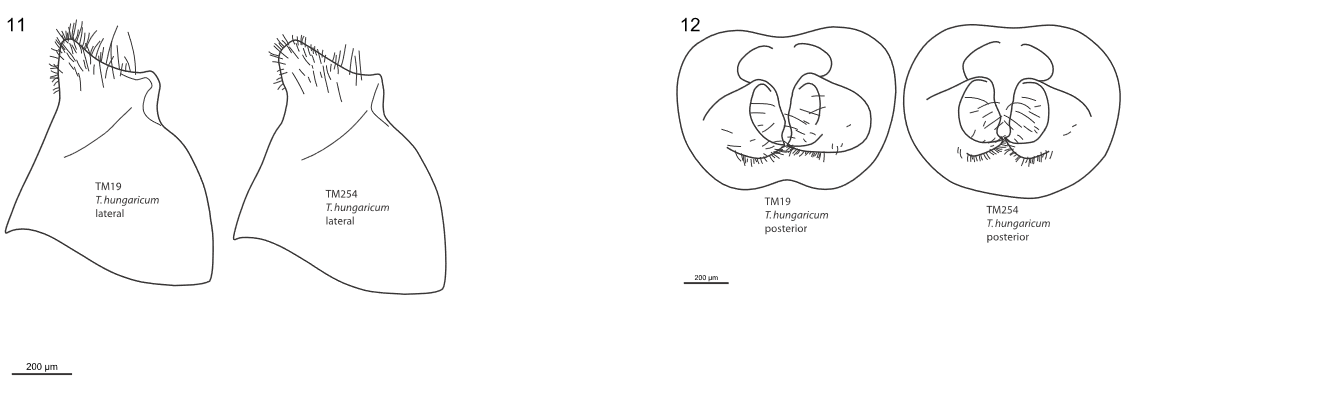


Fig. S5: Male genital morphology of 2 males of *Tetramorium hungaricum* in dorsal, ventral, lateral, and posterior view (ex Wagner et al. 2017).


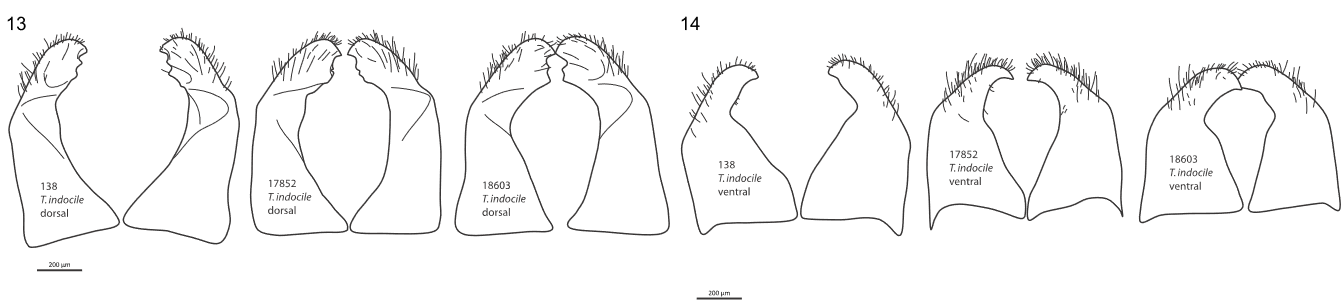

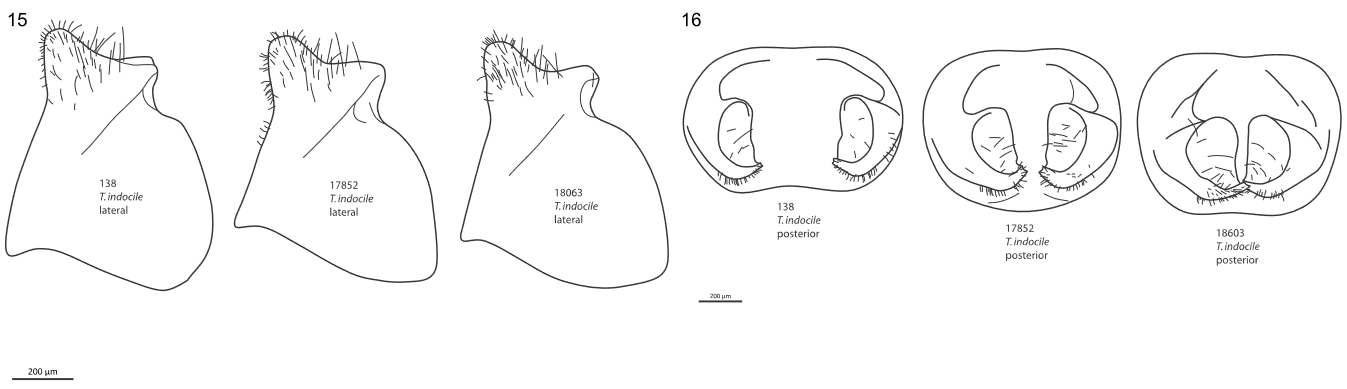
Fig. S6: Male genital morphology of 3 males of *Tetramorium indocile* in dorsal, ventral, lateral, and posterior view (ex Wagner et al. 2017).


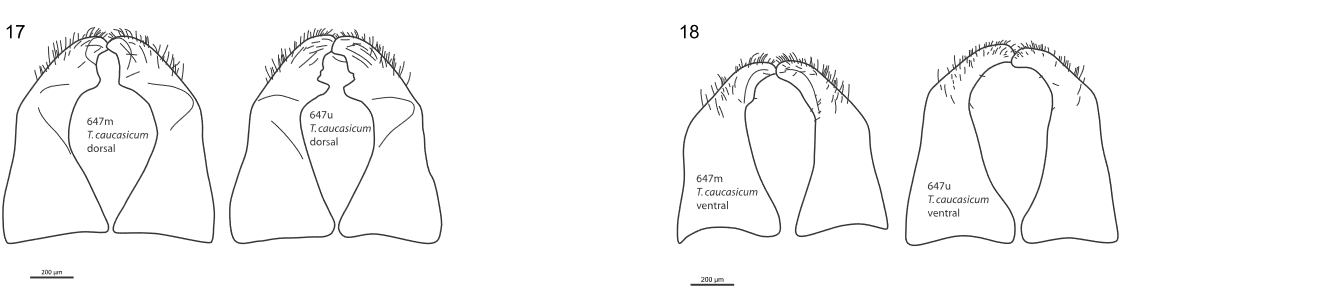


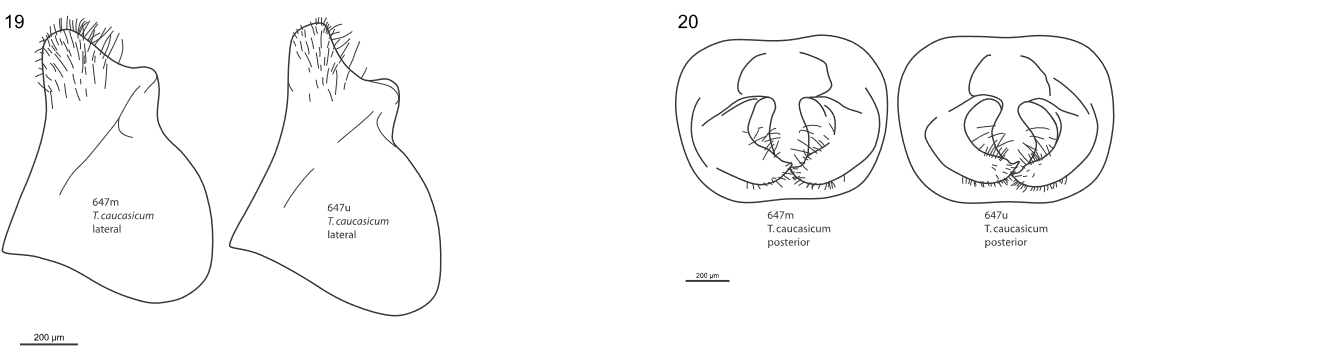
Fig. S7: Male genital morphology of 2 males of *Tetramorium caucasicum* in dorsal, ventral, lateral, and posterior view (ex Wagner et al. 2017).


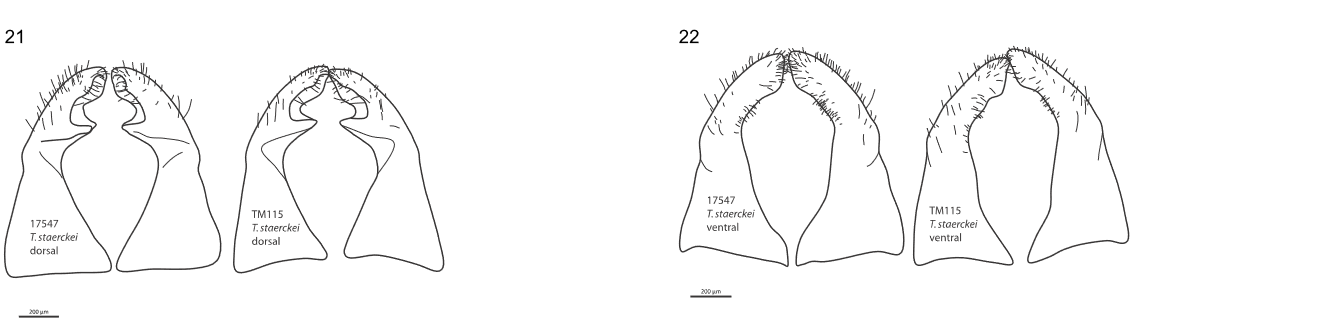

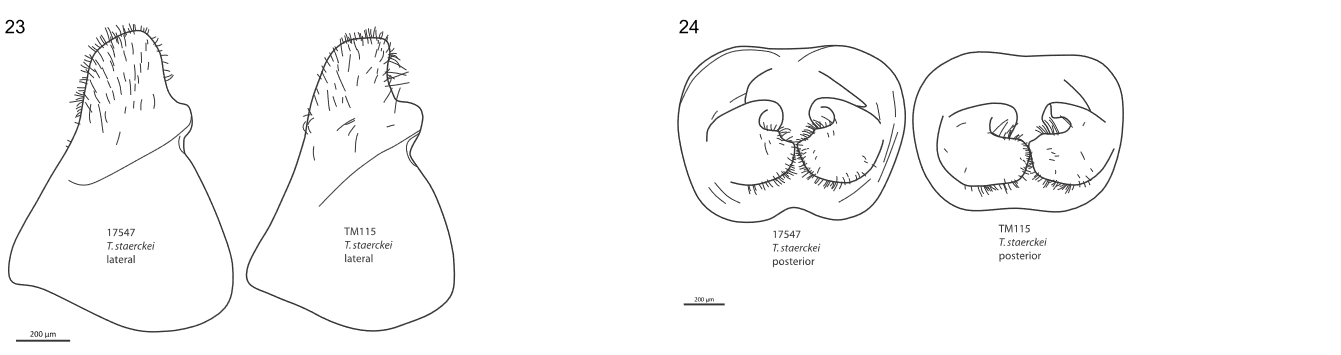


Fig. S8: Male genital morphology of 2 males of *Tetramorium staerckei* in dorsal, ventral, lateral, and posterior view (ex Wagner et al. 2017).


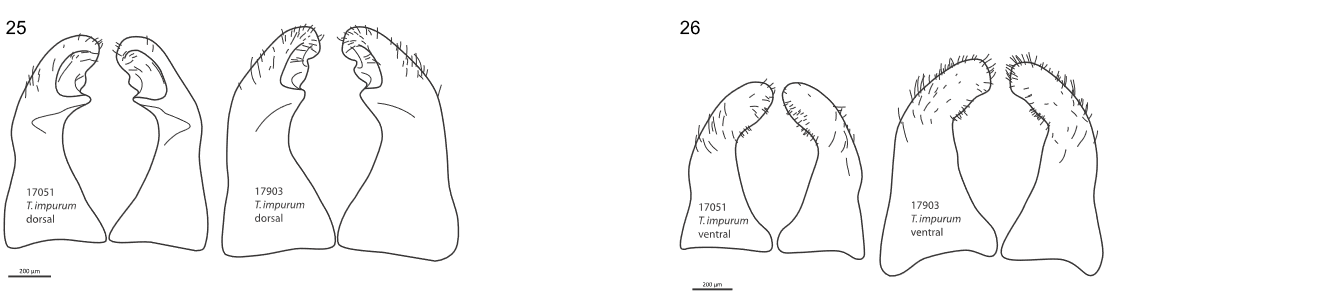

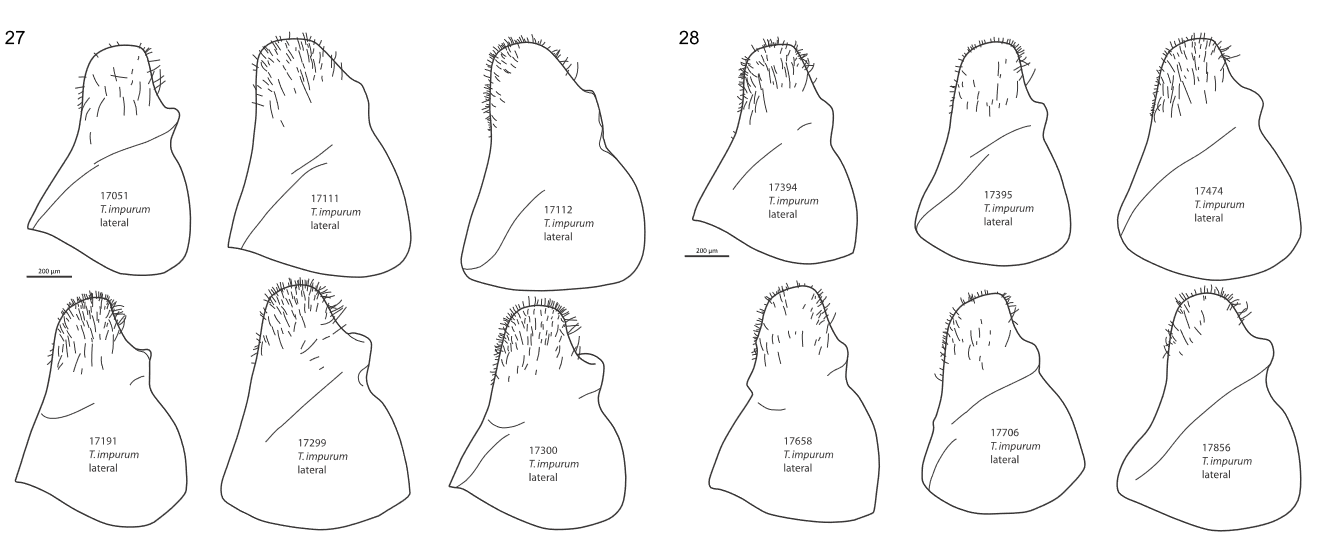

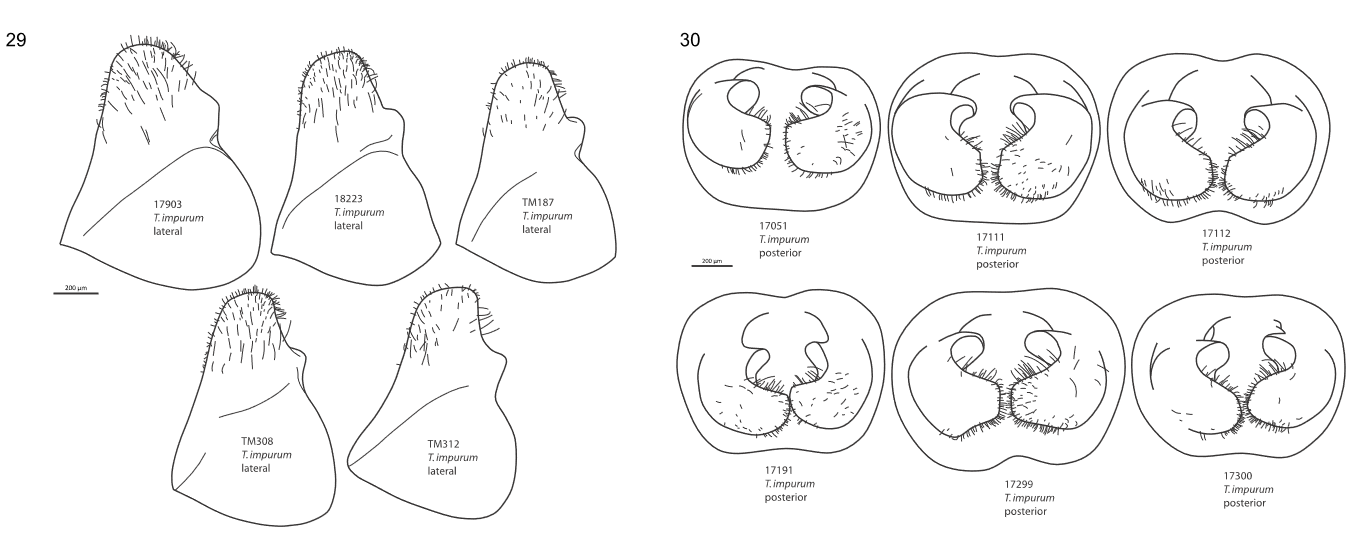


Fig. S9: Male genital morphology of males of *Tetramorium impurum* in dorsal, ventral, lateral, and posterior view (ex Wagner et al. 2017).
