# Supplementary material for: ﻿Delineation of species of the Tetramoriumcaespitum complex (Hymenoptera, Formicidae) in Anatolia with a diagnosis of related species-complexes
Source: Zookeys. 2025 Apr 22;1234:309–39. doi: 10.3897/zookeys.1234.142963 (PMC12041867; doi:10.3897/zookeys.1234.142963)
Supplement: Supplementary material 4 — Observed and expected heterozygosity, the number of alleles, and the effective alleles of microsatellite data [file zookeys-1234-309_article-142963__-s004.docx]

Supplement Figs of

**Delineation of species of the *Tetramorium caespitum* complex (Hymenoptera, Formicidae) in Anatolia with a diagnosis of related species-complexes**

Herbert C. Wagner, Marion Cordonnier, Bernard Kaufmann, Kadri Kiran, Celal Karaman, Roland Schultz, Bernhard Seifert, Sándor Csősz

|  | Genalex | | | | | |
| --- | --- | --- | --- | --- | --- | --- |
|  | N: no. of genotypes, Na: no. of different alleles, ne: No. of effective alleles, ho: observed heterozygosity, he: expected heterozygosity, F: fixation index | | | | | |
| **Locus** | **N** | **Na** | **Ne** | **Ho** | **He** | **F** |
| Tspe52d | 134 | 65 | 36.092 | 0.799 | 0.972 | 0.179 |
| Tspe52b | 106 | 45 | 30.868 | 0.745 | 0.968 | 0.230 |
| Tspe52k | 133 | 30 | 17.769 | 0.722 | 0.944 | 0.235 |
| Ttsu56d | 135 | 23 | 12.402 | 0.815 | 0.919 | 0.114 |
| Tspe53a | 134 | 25 | 13.930 | 0.754 | 0.928 | 0.188 |
| Ttsu55a | 134 | 39 | 29.852 | 0.791 | 0.967 | 0.182 |
| Ttsu59j | 127 | 40 | 19.201 | 0.756 | 0.948 | 0.203 |
| Tspe51i | 134 | 59 | 38.327 | 0.888 | 0.974 | 0.088 |
| Ttsu58i | 134 | 33 | 18.257 | 0.821 | 0.945 | 0.132 |
| Tspe51a | 127 | 43 | 19.538 | 0.709 | 0.949 | 0.253 |
| Tspe51o | 134 | 47 | 27.625 | 0.828 | 0.964 | 0.141 |
| Tspe51b | 120 | 48 | 29.783 | 0.783 | 0.966 | 0.189 |
| Tspe51d | 113 | 31 | 17.290 | 0.779 | 0.942 | 0.173 |
| Tspe52a | 133 | 19 | 13.255 | 0.654 | 0.925 | 0.292 |
| Tspe53b | 135 | 26 | 11.546 | 0.659 | 0.913 | 0.278 |
| Ttsu57l | 127 | 50 | 33.393 | 0.654 | 0.970 | 0.326 |
| Ttsu56h | 133 | 36 | 16.364 | 0.654 | 0.939 | 0.303 |
